# The therapeutic landscape of cannabidiol: a systematic review of molecular mechanisms, clinical efficacy in oncology and neurology, and regulatory changes

**DOI:** 10.1186/s42238-026-00482-0

**Published:** 2026-08-06

**Authors:** Calin Trif, Chiravoot Pechyen, Yardnapar Parcharoen

**Affiliations:** 1 Department of Materials Technology and Textile, Faculty of Science and Technology, Thammasat University, Pathum Thani, 12120 Thailand; 2 Center of Excellence in Modern Technology and Advanced Manufacturing for Medical Innovation, Thammasat University, Thammasat University, Pathum Thani, 12120 Thailand; 3 Chulabhorn International College of Medicine, Thammasat University, Pathum Thani, 12120 Thailand

**Keywords:** Cannabidiol, CBD, Pharmacokinetics, Safety, Clinical efficacy, Legislation

## Abstract

Despite extensive research on CBD, a comprehensive analysis of its rapidly evolving molecular understanding, clinical validation across diverse fields such as oncology and neurology, safety concerns, and dynamic global regulatory changes remain fragmented. This review addresses a critical translation gap by integrating quantitative molecular polypharmacology with the most recent global regulatory thresholds. Furthermore, it evaluates how pharmacogenomic modeling and advanced nanocarrier delivery systems based on artificial intelligence can actively resolve the pharmacokinetic limitations of CBD and dose-dependent hepatotoxicity, establishing a clear, data-driven design for personalized cannabinoid medicine. CBD acts as a broad-spectrum modulator through multiple molecular targets, including cannabinoid and non-cannabinoid receptors, contributing to its pharmacological effects. Clinical evidence strongly supports its efficacy in pediatric seizure disorders and symptom management in oncology, while topical applications show promise in pain and dermatological conditions. However, systemic CBD use for chronic pain, psychiatric disorders, and as a primary anti-tumor agent still requires reliable clinical validation. Regulatory frameworks are changing globally in response to emerging safety concerns, particularly hepatotoxicity and drug interactions mediated by CYP450 inhibition. Future research priorities should include large-scale trials to clarify the inter-entourage effect, long-term safety assessments, metabolic interventions and precision delivery systems. This review highlights the necessity of evidence-based approaches to unlock the full clinical potential of CBD within a controlled, scientific-based approach.

**Supplementary Information:**

The online version contains supplementary material available at https://doi.org/10.1186/s42238-026-00482-0.

## Introduction

*Cannabis sativa* is a fascinating botanical species with a long history of global medicinal and industrial application (Ren et al. 2021; Charitos et al. 2021; Sawler et al. 2015). While historical taxonomy heavily debated the existence of multiple distinct species (such as *Cannabis sativa, indica and ruderalis*) based on physical traits, modern genomic analyses suggest the genus is a highly diverse monotypic species (Lapierre et al. 2023; Small 2015). Regardless of botanical classification, the plant produces a complex matrix of over 125 identified terpenophenolic compounds, or cannabinoids, alongside various terpenoids and flavonoids (Radwan et al. 2021; Fordjour et al. 2023; Laaboudi et al. 2024). Delta-9-tetrahydrocannabinol (THC) and cannabidiol (CBD) represent the primary therapeutic compounds (Ren et al. 2021).

Because visual morphology and physiological parameters are not reliable indicators of the plant chemical composition (Murovec et al. 2022; Smith et al. 2022), modern botanical and pharmaceutical classification relies strictly on chemical profiling. This system separates the plants into five distinct chemotypes based on their primary cannabinoid ratios (Table 1) (Small et al. 1973; Mandolino et al. 2004). Chemotype III, which produces prevalent CBD and minimal THC, serves as the primary agricultural source for modern commercial CBD extraction (Small et al. 1973; Pearce et al. 2014).

**Table 1:** Chemotypes of cannabis

Chemotype	Primary Cannabinoid Profile	Cannabinoids			Cannabinoid ratio
		THC	CBD	CBG	
I	THC-Prevalent (Drug-type)	≥ 0.3%	< 0.5%	-	CBD:THC < 1
II	Intermediate (Co-dominant)	> 0.3%	> 0.5%	-	CBD:THC 1:1—3:1
III	CBD-Prevalent (Fiber/Oil-type)	< 0.3%	≥ 1%	-	CBD:THC ≥ 5:1
IV	CBG-Prevalent	≤ 0.3%	< 0.5%	> 1%	CBG > 50%
V	No cannabinoids	Total cannabinoids ≤ 0.1%			Trace cannabinoids

Over the past decade, clinical focus has narrowed significantly onto highly purified CBD isolates (Gloss 2015). The regulatory approval of Epidiolex in 2018 for treatment-resistant epilepsy syndromes marked the transition of cannabinoids from unauthorized alternative treatments to strictly standardized, prescription-only active pharmaceutical ingredients (Small 2015, 2017; Aggarwal et al. 2009). Individual phytocannabinoids that received marketing authorization are CBD (Epidiolex™), THC (INN: dronabinol, Marinol™) and a combination of two refined extracts (nabiximols, Sativex™). In turn, the market now contains a handful of OTC products containing cannabinoids, isolated or full spectrum. While CBD demonstrates profound efficacy for specific indications, such as refractory pediatric epilepsy, it often does not outperform placebos in many other highly publicized use cases, including general chronic pain, clinical depression, and generalized anxiety (Cásedas et al. 2024). Furthermore, the assumption that CBD is a biologically inert, universally safe supplement is contradicted by increasing pharmacological research demonstrating dose-dependent hepatotoxicity and severe drug-drug interactions, particularly those mediated through the hepatic cytochrome P450 enzyme system (Smith et al. 2022).

**Fig. 1:**
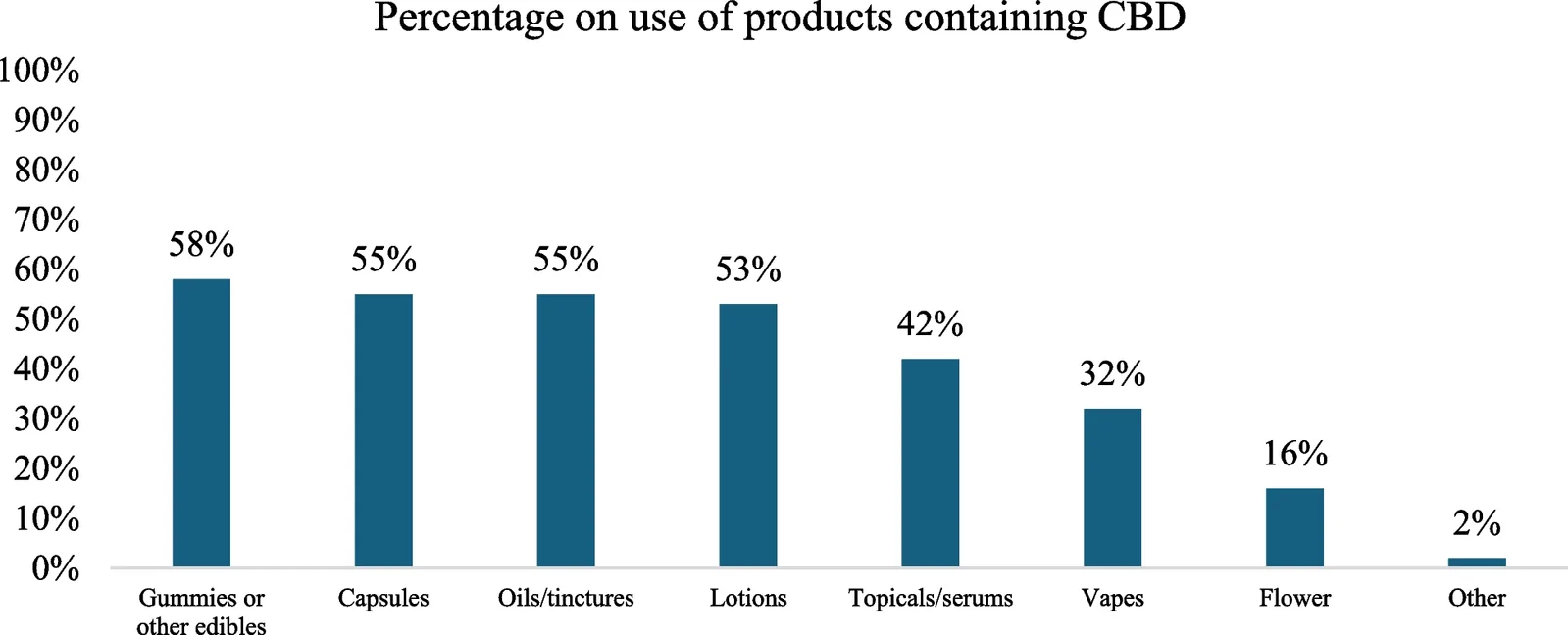
Consumption of cannabidiol products among U.S. population. Data represent descriptive percentage frequencies for a consumer survey of U.S. adults tracking public perception, common reasons for use (stress reduction, sleep quality, and pain relief), and preferred product modalities. Percentages illustrate a descriptive view of the consumer supplement market across states where hemp-derived CBD products containing less than 0.3% THC are legal (Hall 2024)

Public perception has also changed since many countries have decided to legalize the use of cannabis, either medical or recreational. Products containing CBD have gained significant public interest and widespread use in recent years, with studies showing varying levels of adoption across different countries. In Canada and the United States, past 12-month CBD product use was reported at 16.2% and 26.1%, respectively (Goodman et al. 2022). Over half of CBD consumers believed CBD oil was beneficial for health. According to a health survey of individuals consuming CBD in the U.S. the most common products were gummies or other edibles, followed by capsules and oils (Hall 2024) (Fig. 1). However, since these surveys are subject to self-reporting sampling bias, the inherent limitations of web-based polling, and significant regional variability in cannabis legality, they should be interpreted with caution and should not be extrapolated.

In another study done in Germany, 40.2% of participants were aware of CBD products, with 11.4% having used them (Geppert et al. 2020). More than half of the consumers perceived the health benefits of CBD use as high or very high, while most rated the health risks as low or very low. The public perception of CBD is generally positive, with many users and potential users viewing it as a natural and safe alternative for managing various health conditions.

Currently, the regulatory situation of cannabis is experiencing a global rearrangement. Jurisdictions that previously supported unregulated cannabis decriminalization, most notably Thailand, have introduced severe legislative reversals in 2025 and 2026 to recapture the market under a strict, medical-only framework (Thailand Cannabis Regulation 2025; Hassan et al. 2025). Alongside, European authorities have drastically reduced the provisional safe intake levels for novel CBD foods, warning for a heightened awareness of long-term toxicological risks and developmental hazards (N.F. EFSA Panel on Nutrition 2026). Given the rapid commercialization, evolving regulatory landscape, and the emergence of new technologies such as AI and nanotechnologies, existing literature often lack a timely, integrated perspective (Vitetta et al. 2025). To address this fragmented landscape, this systematic review provides a new, translational synthesis that bridges the gap between preclinical hypotheses and human clinical results. Additionally, this review correlates standardized, quantitative receptor kinetics with recent 2026 regulatory standards, including the EFSA novel food mandates and Thailand's legislative reversals. Significantly, this review advances the current literature by critically evaluating how computational high-throughput screening, AI-driven pharmacogenomic risk stratification, and advanced nanocarrier delivery systems can overcome the systemic bioavailability barriers of CBD and CYP450-mediated hepatotoxicity, thereby defining the future parameters of evidence-based cannabinoid therapeutics.

## Methodology

### Search strategy

**Fig. 2:**
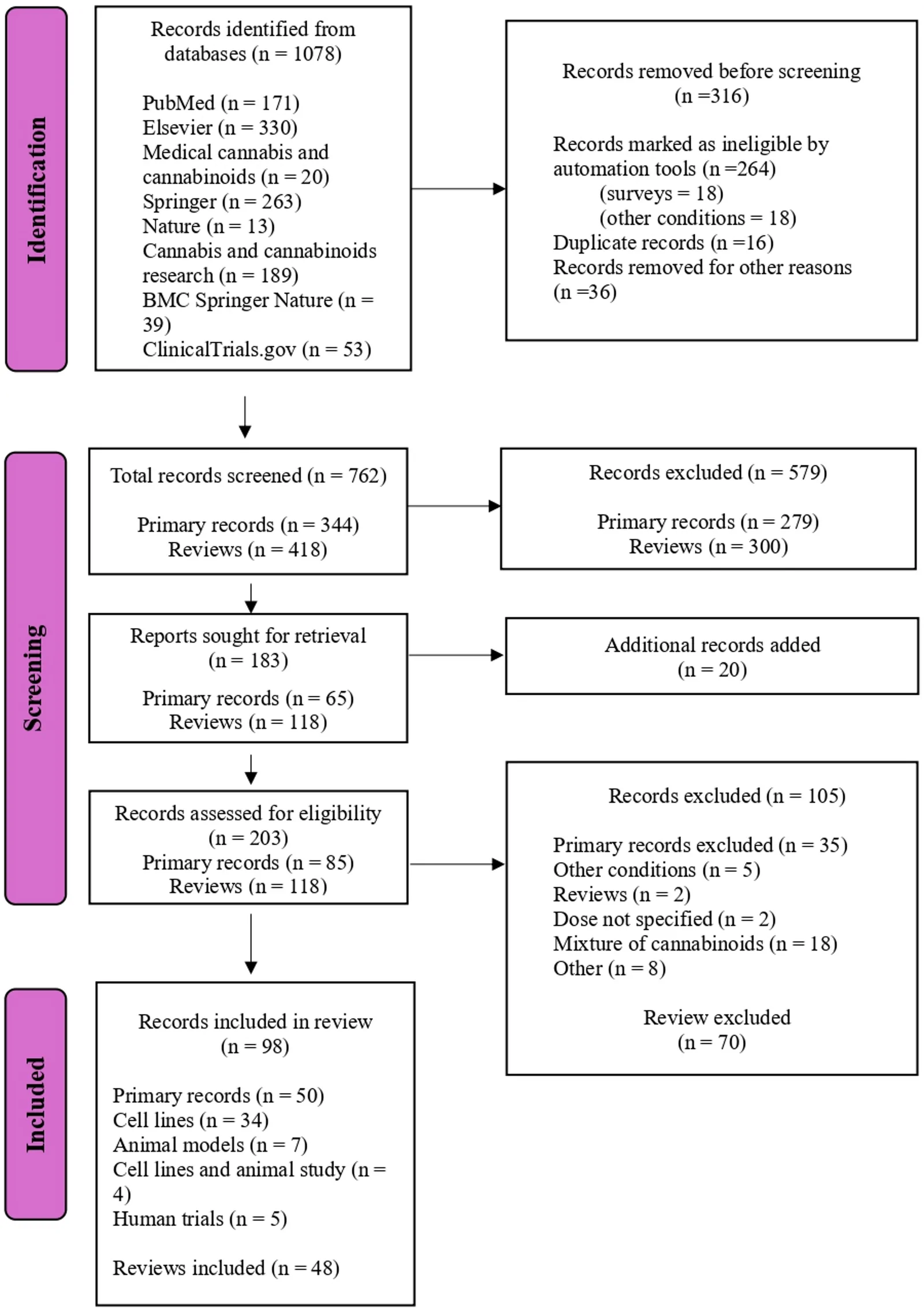
PRISMA diagram

To identify relevant studies, we strategically searched a combination of broad medical and specialized cannabis-focused databases (PubMed, Elsevier, Springer, Nature, ClinicalTrials.gov, and specific journals (Medical Cannabis and Cannabinoids, Cannabis and Cannabinoids Research, BMC Springer Nature) to ensure best coverage in the therapeutic landscape of CBD. The search strategy, shown in the PRISMA diagram below (Fig. 2) involved the use of the following keywords “hemp”, “cannabis”, “CBD”, “cancer”, “clinical”. The literature published from January 1, 2004, through January 31, 2026, was analyzed. The initial search return yielded a total of 1,078 articles. Language restrictions were applied to filter exclusively for peer-reviewed studies published in English.

### Inclusion and exclusion criteria

To achieve a comprehensive and focused synthesis, we established two primary inclusion directions: (1) randomized controlled trials (RCT) specifically investigating isolated CBD for cancer management, published up to the search date (last 20 years), ensuring a focus on high-level evidence, and (2) systematic reviews and meta-analyses published within the last five years focusing on the use of CBD for human health, including oncology and other neurological or dermatological conditions as detailed in the results. The primary records search had yielded a significantly low number of articles, since most of the clinical trials had not published their results. Records were strictly excluded if they consisted of unstandardized consumer surveys, evaluated mixed cannabinoid formulations without a quantified, declared CBD:THC content, or comprised conference abstracts and case reports with sample sizes smaller than 5.

### Quality assessment and synthesis of cannabinoid formulations

Data from all included studies were extracted into a pre-defined template covering study design, participant characteristics, interventions, outcomes, and key findings. Review articles were excluded if they were not systematic reviews or meta-analyses, or if they were published more than five years prior to the search date, to ensure recent coverage and methodological accuracy.

To control the high heterogeneity of evaluated preparations (purified CBD isolates vs. full-spectrum extracts), data extraction templates explicitly ranked compounds by formulation purity, delivery method, and specific dose thresholds. Risk-of-bias assessments were systematically applied by two independent investigators. Preclinical in vivo animal models were assessed using the SYRCLE risk-of-bias tool (Hooijmans et al. 2014). Human clinical trials and primary RCTs were examined using the Cochrane Risk of Bias (RoB 2) methodology (Sterne 2019) to judge randomization, protocol deviations, missing outcome parameters, and measurement bias (supplementary material).

## Results

### Molecular mechanisms and pharmacodynamics

The endocannabinoid system (ECS) is an important lipid signaling network with a fundamental role in maintaining physiological homeostasis (Lu et al. 2016; Grether 2025). The ECS includes endogenous lipid ligands (endocannabinoids such as anandamide and 2-arachidonoylglycerol), their biosynthetic and hydrolytic enzymes (such as FAAH), and the G protein-coupled cannabinoid receptors, primarily CB1 and CB2 (Bayewitch et al. 1996; Kopustinskiene et al. 2022; Behl et al. 2022). Dysregulation of this system is heavily implicated in metabolic disorders, cardiovascular diseases, and neurodegenerative conditions (Grether 2025). While THC acts as a potent partial agonist at both CB1 (highly concentrated in the central nervous system) and CB2 (localized primarily on immune cells), CBD possesses a different pharmacological profile (Grether 2025; Bayewitch et al. 1996; Kopustinskiene et al. 2022). CBD exhibits low binding affinity for both classical cannabinoid receptors (Singh et al. 2026). Instead of direct agonism, CBD acts as a negative allosteric modulator of CB1 (Cásedas et al. 2024). This allosteric modulation is the primary mechanism by which CBD attenuates the psychoactive, anxiogenic, and tachycardic adverse effects of THC when the two compounds are co-administered, effectively altering the receptor conformation to reduce binding efficacy (Cásedas et al. 2024).

Apart from the classical ECS, the broad therapeutic profile of CBD is achieved through a diverse range of non-cannabinoid receptors, ion channels, and molecular pathways (Table 2). A recent review by Białoń M. et all highlighted that CBD exhibits pathway selectivity, particularly at the 5-HT1A receptor, where it may preferentially activate specific intracellular cascades over others, favoring G-protein activation while showing minimal beta-arrestin recruitment (Białoń et al. 2026).

**Table 2:** Molecular mechanisms of CBD

Molecular Target	Mechanism of Interaction	Binding Affinity/Potency	Primary Dose-Dependent Physiological Impact	Ref
CB1	Negative allosteric modulator (high non-competitive selectivity)	K_i_ ≈ 10–15 µM	Attenuates THC psychoactive, anxiety-inducing, and tachycardic adverse effects when co-administered and increases neurogenesis	Cásedas et al. 2024)
CB2	Low-affinity inverse agonist/antagonist	K_i_ > 10 µM	High dose (300 mg/day) suppresses immune cell activation; downregulates pro-inflammatory cytokine cascades in peripheral tissues	Grether 2025; Bayewitch et al. 1996)
5-HT1A Receptors	Direct partial agonist (exhibits high pathway selectivity)	K_i_ ≈ 16 µM	Low dose promotes daytime wakefulness via somatodendritic autoreceptor activation High dose triggers postsynaptic cascades to drive anxiolytic and anti-neuropathic effects	Singh et al. 2026)
TRPV1 Channels	Full agonist followed by rapid desensitizer	EC_50_ ≈ 1–3 µM	Biphasic action, acute low doses trigger transient burning/nociception; sustained higher doses induce profound calcium channel desensitization, causing analgesia	Singh et al. 2026)
PPARγ Receptors	Nuclear receptor agonist	EC_50_ ≈ 5 µM	Sustained dosing crosses the nuclear membrane to inhibit NFκB transcriptional pathways; blocks downstream neuroinflammatory gene expression	Aychman et al. 2023)
GPR55 & GPR18	Direct competitive antagonist	K_i_ ≈ 0.445 µM	Low-moderate dose blocks endogenous lysophosphatidylinositol (LPI) binding; stabilizes intracellular calcium modulation to mitigate seizure activity	Singh et al. 2026)
Dopamine D2	Partial agonist (functional selectivity)	K_i_ ≈ 12.8 µM	High dose modulates dopaminergic pathways, contributing to the biphasic sedative transition and antipsychotic properties seen in early trials	Cásedas et al. 2024)
Adenosine A2A	Indirect enhancer (via competitive inhibition of the ENT1 transporter)	IC_50_ ≈ 0.250 µM	Low-moderate dose accumulates extracellular adenosine, amplifying endogenous A2A signaling to shift microglia from pro-inflammatory (M1) to repair (M2) states	Aychman et al. 2023)
FAAH Enzyme	Indirect competitive inhibitor	IC_50_ ≈ 2.5 µM	Dose-Proportional progressively blocks anandamide (AEA) breakdown, elevating endogenous homeostatic signaling to promote deep slow-wave sleep	Białoń et al. 2026)
FKBP5 Protein	Functional antagonist (downregulates chaperone expression)	Validated via downstream protein expression assays	Sustained high dose restricts immunophilin chaperone activity, effectively blocking glucocorticoid receptor resistance and NF-κB-driven inflammatory signaling cascades	Białoń et al. 2026)

While these complex receptor-level interactions outline a complex polypharmacological profile in laboratory conditions, a significant translational disconnect continues regarding human clinical validation. The vast majority of these molecular pathways, specifically negative allosteric modulation of CB1, GPR55 antagonism, and competitive FAAH enzyme inhibition, have been validated exclusively in in vitro expressions or animal models. Clinically validated mechanisms in human subjects remain strictly limited to specific neuro-structural channels controlling seizure moderation, leaving the larger systemic receptor pathways characterized as exploratory therapeutic hypotheses rather than established clinical facts.

### Pharmacokinetics and advanced delivery systems

The pharmacokinetics of CBD are highly variable and heavily dependent on the route of administration, representing a major difficulty for clinical consistency (Paczkowska-Walendowska et al. 2025). Oral administration, which accounts for the vast majority of consumer use via oils, capsules, and gummies, has a major drawback because of extensive first-pass hepatic metabolism and the compound high lipophilicity (Paczkowska-Walendowska et al. 2025). Consequently, the absolute oral bioavailability of CBD is remarkably low, estimated at only 9% to 13% (Żółnowska 2025). Because of this poor absorption, orally administered CBD has a prolonged time to maximum plasma concentration (*T*_*max*_) and requires significantly higher doses to achieve therapeutic blood levels compared to inhalation or transdermal delivery methods (Cásedas et al. 2024). This pharmacokinetic issue frequently makes low-dose commercial supplements (for example 10 mg gummies) biologically inactive on a systemic level, suggesting these low-dose formulations may lack systemic pharmacological effect. Pharmacological development has turned toward new nanotechnologies to overcome these challenges, and they are shown in Table 3.

**Table 3:** Nanotechnologies for improving the bioavailability of CBD

Delivery method	Technology	Bioavailability	T_max_	Absorption	Limitations	Ref
Standard oral oil/capsules	Encapsulation	9–13%	120–300 min	Intestinal enterocyte uptake followed by extensive hepatic first-pass CYP450 clearance	High inter-patient variability; highly dependent on dietary fat co-ingestion to achieve therapeutic minimums	Paczkowska-Walendowska et al. 2025; Żółnowska 2025)
Nanocarriers/ Nanoemulsions	Lipid-based encapsulation	22–40%	45–90 min	Bypasses portal circulation via uptake into the intestinal lymphatic system	Long-term physical stability of sub-micron droplets; industrial scale-up constraints	Paczkowska-Walendowska et al. 2025; Afonso et al. 2026; Theo 2025)
CBD-IN (Intranasal/Inhaled)	Water-soluble nano-micelles	45–60%	10–30 min	Direct olfactory region transmucosal diffusion; targeted nose-to-brain pathway	Mucosal irritation limits; strict restriction on total fluid volume delivery per spray event	Feng et al. 2025)
Cyclodextrin inclusion	Molecular complexes	25–34%	30–60 min	Hydrophilic exterior allows rapid dissolution in the aqueous gastric boundary layer	High molecular excipient weight ratio requirements; localized gastric wall tolerability at high doses	Paczkowska-Walendowska et al. 2025)
Micro-needle TDDS	Transdermal delivery	30–45%	Continuous release	Mechanical disruption of the *stratum corneum* to access dermal capillaries directly	Micro-needle structural mechanics and patch skin-adhesion durability under chronic moisture conditions	Feng et al. 2025)

**Fig. 3:**
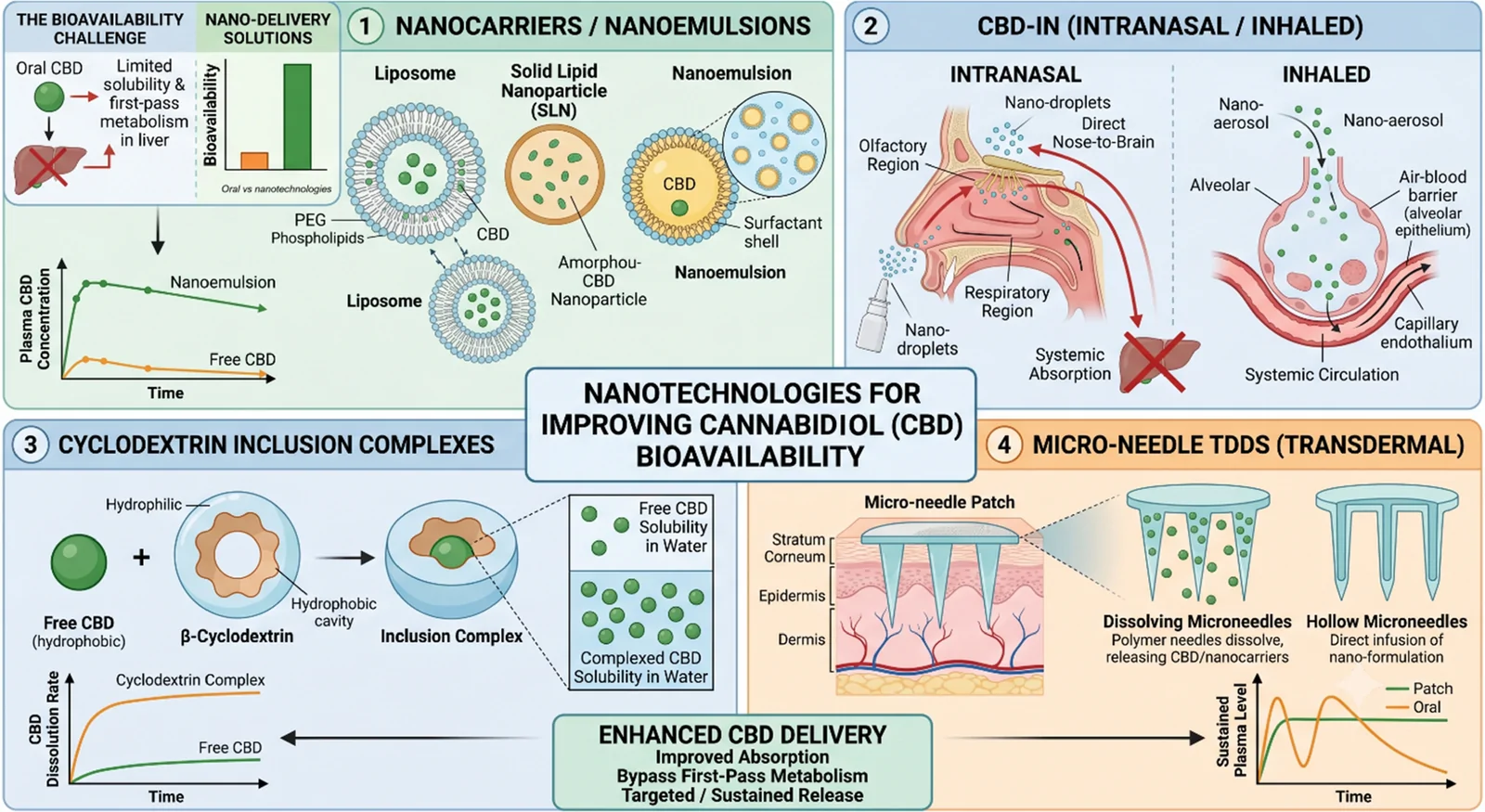
Mechanistic actions and translational pathways of advanced nanotechnology delivery systems for CBD. The illustrations trace how engineered matrices actively overcome the primary biological barriers to cannabinoid absorption (poor water solubility and heavy hepatic first-pass clearance). (1) Nanoemulsions exploit lymphatic transport to increase plasma concentrations. (2) Intranasal micelles exploit mucosal diffusion for rapid, nose-to-brain therapeutic effects within 30 min. (3) Cyclodextrin complexes enclose the hydrophobic cannabinoid core in a hydrophilic shell to accelerate dissolution speeds. (4) Dissolving or hollow transdermal microneedles pierce outer skin barriers to establish sustained, steady-state blood plasma levels. Translational success depends on optimizing formulation physical stability, ensuring precise particle scale-up uniformity, and modifying localized tissue encapsulation or site irritation

The shift in nanomedicine research is moving past simple drug carriage toward intelligent, mechano-responsive therapeutics (Vitetta et al. 2025; Onaivi et al. 2020), as shown in Fig. 3. Real-time Critical Quality Attribute (CQA) monitoring, supported by artificial intelligence, is another technology being introduced on production lines to ensure precise particle size distribution in CBD nano-formulations, reducing the uncertainty previously seen in cannabinoid therapeutics (Wasalathanthri et al. 2020). CBD mixed with oils and other active ingredients has been studied as well for specific applications while exploring its stability, bioavailability, and therapeutic potential (Kosović et al. 2021).

### Neurological disorders

The most studied and scientifically validated clinical application of highly purified CBD is in the field of pediatric and adult neurology. The approval of Epidiolex marked a significant milestone for cannabinoid therapeutics. It is currently indicated for the treatment of severe, treatment-resistant seizure disorders, specifically Lennox-Gastaut syndrome (LGS), Dravet syndrome (DS), and tuberous sclerosis complex (TSC) (US_FDA 2026). The clinical efficacy in this domain is proven and statistically significant. A recent review article by Oliviera et. all of large-scale trials demonstrated that patients treated with CBD experienced an average 41.1% reduction in seizure frequency, compared to an 18.1% reduction among those receiving a placebo (Oliveira et al. 2025). This represents a 127% higher efficacy rate for the CBD cohort.

As of 2026, the neurological focus has expanded toward autism spectrum disorder (Cazares et al. 2026). A Phase II double-blind, placebo-controlled crossover trial published in 2024 and analyzed through 2026 investigated 24 young males with high-support autism aged 7–14 (Cazares et al. 2026). The findings established a clear pharmacokinetic-pharmacodynamic relationship, which is that measurable blood levels of CBD metabolites correlated directly with broadband EEG changes and functional improvements in visuomotor and non-verbal cognitive abilities. This focus on children with more severe symptoms fills a critical gap in the research, although the authors emphasized that behavioral core symptoms often remain unchanged in short-term trials.

Neuroprotection in the context of cognitive aging has also emerged as a significant research priority (Binkowska et al. 2025). CBD exhibits potent antioxidant capabilities, reducing reactive oxygen species (ROS) and shifting microglia from a damage-causing pro-inflammatory (M1) state to a repair-focused anti-inflammatory (M2) state (Aychman et al. 2023; Bhunia et al. 2022). In longitudinal murine models evaluated in 2025, aging mice administered oral CBD demonstrated significant reductions in brain inflammation and improvements in memory processes mediated by the hippocampus and perirhinal cortex (Mirza Agha et al. 2025). Furthermore, recent studies showed that CBD and CBG together could reduce neuroinflammation markers in Alzheimer models, potentially slowing the progression of neurodegenerative atrophy (Wu et al. 2025). However, despite these studies in animals, human trials for cognitive preservation in healthy older adults are still far from proving the same results as in the murine models (Binkowska et al. 2025).

Despite the profound, statistically significant success of purified CBD in refractory epilepsy (LSG and DS), extending these conclusions to other neurological domains remains experimental. Clinical data for autism spectrum disorder are restricted to very small, short-term crossover pilots (such as the 24-subject cohort mapping baseline EEG shifts), which fail to establish long-term behavioral modifications. Similarly, neuroprotective and anti-inflammatory applications for Alzheimer’s disease are derived entirely from short-term longitudinal murine models (aging C57 mice). Human trials for cognitive preservation in healthy older adults has produced highly inconsistent outcomes and drawing definitive clinical conclusions from these early-stage, animal-centric discoveries introduces relatively high overinterpretation bias.

### Oncology, preclinical findings and clinical evidence

The role of hemp-derived CBD in cancer management is a great interest in modern research, characterized by consistent preclinical results and on-going clinical progress and challenges (Mangal et al. 2021; Heider et al. 2022; O’Brien 2022). While oncology was one of the first areas where clinicians recognized that cannabinoids could be helpful for symptoms management, the search for a direct anti-tumor role continues to lead the search.

This review provides a summary of studies that have investigated the effects of CBD on various types of cancer. These studies comprised a wide range of research methodologies, including preclinical and clinical investigations. Preclinical studies include in vitro experiments using cancer cell lines, while in vivo studies using animal models. Clinical studies included randomized controlled trials, open-label trials, and pilot studies.

#### Preclinical anti-tumor efficacy

Preclinical studies consistently demonstrated the anti-cancer potential of CBD across various cell lines and animal models. CBD exerts its effects through multiple, highly interconnected mechanisms that inhibit cancer cell proliferation and survival. The main findings are shown in Table 4. An extensive table can be found in the supplementary material, Tables S1 and S2 with all studies on CBD cell lines and animal models included in this review. To systematically organize the preclinical findings, Table 4 categorizes the molecular mechanisms of CBD across four core cellular mechanisms. The category dedicated to growth pathways tracks the downregulation or deactivation of molecular proliferation mechanisms, such as mTOR, AKT, or EGFR signaling, that induce cancer cells into functional cell cycle arrest. Another mechanism for apoptosis induction details the upregulation of essential pro-apoptotic regulators, including Caspases, Bax, and p53, which are observed to actively promote programmed cellular death. The final two categories define intracellular stress profiles and the inhibition of metastatic progression. Under cellular stress, the matrix outlines the generation of intracellular oxidative stress, typically driven by a severe accumulation of reactive oxygen species (ROS), calcium (Ca^2^⁺) homeostasis disruption, or acute endoplasmic reticulum (ER) stress. Lastly, the tumor spread evaluates the localized suppression of cancer cell motility, basement membrane tissue invasion, and the downstream inhibition of the angiogenesis required for neo-vascularization.

**Table 4:** CBD mechanism of action in preclinical cancer studies

Cancer Type	Study Type	Scientific Evidence	Outcome	Ref
Breast	In vitro cell lines (MDA-MB-231) & In vivo mouse xenografts	Preclinical Validation	Observed to induce G1-phase cell cycle arrest; upregulates Caspase-3, p53, and Bax; increases ROS and intracellular calcium in models	Ligresti et al. 2006; Fraguas-Sánchez et al. 2020; Patel 2021; Surapaneni et al. 2022; D’Aloia et al. 2022; Śledziński et al. 2023)
Glioma/GBM	In vitro (T98G, U87MG) & In vivo orthotopic syngeneic models	Preclinical Validation	Associated with decreased glioma stem cell (GSC) viability via ROS production; triggers stress granule (TIAR-1) up-regulation	Ligresti et al. 2006; Scott et al. 2014; Singer et al. 2015; Gross et al. 2021; Anceschi et al. 2022; Wang et al. 2024)
Prostate	In vitro cell lines (DU-145, PC-3, LNCaP)	Exploratory/In Vitro Only	Demonstrated a capacity to downregulate androgen receptor (AR) and TRPM8 mRNA; activates Caspase 3/7 pathways in vitro	Ligresti et al. 2006; Śledziński et al. 2023; Petrocellis et al. 2013)
Colorectal	In vitro cell cultures & In vivo mouse carcinogenesis models	Preclinical Validation	Deactivates the mTOR/S6 signaling pathway; shows potential to attenuate colon carcinogenesis by modulating macrophage distributions	Ligresti et al. 2006; Anceschi et al. 2022; Romano et al. 2014; Macpherson et al. 2014; Raup-Konsavage et al. 2018; Sainz-Cort et al. 2020; Sun et al. 2023; Raup-Konsavage et al. 2020)
Leukemia/Lymph	In vitro lymphoblastoid cell lines (Jurkat, HL60, K562)	Exploratory/In Vitro Only	Triggers mitochondrial calcium overload and mPTP opening; interacts with EFTUD2 targets to impair cell line proliferation	Ligresti et al. 2006; Scott et al. 2014; Anceschi et al. 2022; Kalenderoglou et al. 2017; Olivas-Aguirre et al. 2019; Mazuz et al. 2020; Ceccacci 2024)
Neuroblastoma	In vitro (SK-N-SH) & In vivo immunodeficient models	Preclinical Validation	Induces sub-G1 cell accumulation and cleaves Caspase-3; observed to restrict tumor invasiveness in mouse series	Fisher et al. 2016)
Cervical	In vitro cell lines (SiHa, HeLa, ME-180)	Exploratory/In Vitro Only	Associated with ATP depletion, p53/Bax upregulation, and down-regulation of anti-apoptotic proteins (Bcl-2, RBBP6) in vitro	Lukhele et al. 2016; Zhu et al. 2021; Razlog et al. 2023)
Pancreatic	In vitro lines (AsPC-I, PANC-I) & In vivo KPCG mice	Preclinical Validation	Inhibits GPR55 signaling to reduce MAPK pathways; upregulates ceramide synthase 1 (CerS1) to prompt ER stress in mouse tissue	Ferro et al. 2018; Mangal et al. 2024)
Endometrial	In vitro endometrial cell lines (Ishikawa, Hec50co)	Exploratory/In Vitro Only	Prompts a reduction in cell line viability through TRPV1 activation and subsequent cleaved PARP elevation in vitro	Fonseca et al. 2018)
Liver (HCC)	In vitro hepatoma cell cultures (HepG2, Huh-7)	Exploratory/In Vitro Only	Demonstrates dose-dependent mitochondrial-dependent pathway activation (BAX, Caspases-3/8/9) inside localized cell models	Zhu et al. 2021; Tajik et al. 2022; Stern et al. 2024)
Bladder	In vitro human transitional carcinoma lines (T24)	Exploratory/In Vitro Only	Associated with cell line viability reductions and decreased cell invasion markers across isolated Matrigel assays	Tomko et al. 2021; Whynot et al. 2023)
Gastric	In vitro epithelial gastric cell lines (AGS)	Exploratory/In Vitro Only	Impairs DNA replication and induces localized mitochondrial stress markers under varying serum concentration parameters in lab settings	Ligresti et al. 2006; Sainz-Cort et al. 2020)
Other*	In vitro cell cultures & In vivo murine models	Mixed Preclinical/Exploratory	Interrupted EGFR-TK signaling pathways in vitro; associated with increased BAX expression and cytochrome c release; demonstrated preliminary capacity to suppress localized tumor growth in early animal configurations	Ligresti et al. 2006; Fraguas-Sánchez et al. 2020; Zhu et al. 2021; Rajan et al. 2016; Maguire et al. 2021; Andradas et al. 2021; Lamtha et al. 2022; Pongking et al. 2024)

^*^includes thyroid (Ligresti et al. 2006), macrophage (Rajan et al. 2016), ovarian (Fraguas-Sánchez et al. 2020; Maguire et al. 2021), medulloblastoma/ependymoma (Andradas et al. 2021), lung (Zhu et al. 2021; Lamtha et al. 2022), and cholangiocarcinoma (Pongking et al. 2024)

In a study by Moholkar et all. specific innovation in delivery methods for oncology have been reported, where exosome-based oral CBD formulations significantly improved tumor targeting and slowed the growth of aggressive triple-negative breast cancer in mice by altering the activity of more than 1,000 genes tied to cancer progression (Moholkar et al. 2026). Similarly, poly-ε-caprolactone microparticles loaded with CBD and THC have shown sustained release over several days, successfully reducing tumor growth in glioma xenograft models (Fraguas-Sánchez et al. 2020).

#### Clinical evidence for symptom management and palliative care

Despite these promising localized effects, standardized randomized human trials have not yet demonstrated clear clinical anti-tumor benefits in patients. The current clinical utility of CBD in oncology is primarily limited to the management of cancer-related symptoms and chemotherapy side effects. Below is a summary table of the studies investigating CBD for cancer management (Table 5). An extensive table with more detailed information can be found in the supplementary material, Tables S3.

**Table 5:** Summary of clinical trials investigating CBD in cancer symptoms management

Cancer Type (Year)	Subjects (n)	Symptom Distress	Inflammation	Drug Interactions & Side Effects	Taste & Physical Function	Ref No
Advanced Cancers (2020)	21 subjects (16 CBD, 5 THC)	↓ Marked improvement in total symptom distress	-	Well-tolerated at a median dose of 300 mg/day	-	Good et al. 2020)
Advanced Cancers (2022)	144 subjects (72 per arm)	** → ** No statistically significant improvement vs. placebo	-	Median daily dose tested at 400 mg	-	Hardy et al. 2023)
Advanced Cancers (2023)	141 subjects (71 placebo, 70 CBD)	-	** → ** No detectable anti-inflammatory effect	Escalating doses up to 600 mg/day	-	Gurgenci et al. 2023)
Breast Cancer (ER +) (2023)	26 subjects	-	-	** → ** No significant impact on tamoxifen levels ↓ May improve tamoxifen side effects	-	Buijs et al. 2023)
Colorectal, Uterine, Ovarian (2023)	32 subjects (22 intervention, 10 control)	-	-	Tested during active chemotherapy cycles	↑ Gained ability to significantly differentiate sweetness (p = 0.03) and saltiness (p = 0.04)	Dominiak et al. 2023)

The analysis of the clinical trials included in this review showed mixed results. In advanced cancer patients, medicinal cannabis trials were found to be generally well-tolerated, with some indications of improvement in emotional and stress-related measures. However, a major randomized placebo-controlled study (MedCan1-CBD) concluded that CBD oil did not provide additional benefits in reducing total symptom distress compared to specialist palliative care alone (Hardy et al. 2023). A statistically significant improvement in ESAS pain scores was noted in some mixed THC:CBD arms, but often at the expense of greater psychomimetic toxicity (Hardy et al. 2025). On the other hand, targeted symptom relief shows promise. A pharmacokinetic study of tamoxifen in breast cancer patients reported that CBD oil use did not significantly alter endoxifen levels, easing concerns about drug-drug interactions with this critical oncology medication, while simultaneously suggesting improvements in endocrine symptoms and quality of life (Gurgenci et al. 2023). Furthermore, pilot trials have indicated that CBD may improve patients ability to differentiate taste strengths during chemotherapy, potentially addressing the debilitating taste alterations that contribute to malnutrition in cancer patients (Buijs et al. 2023; Dominiak et al. 2023).

Notably, a certain distinction must be maintained between palliative symptom management and curative anti-tumor interventions. While preclinical models show a selection of anti-cancer mechanisms, there is currently no convincing, high-level human clinical trial evidence demonstrating that purified or full-spectrum CBD can function as a primary anti-tumor agent for cancer in human patients. Implying direct anti-cancer interpretation threatens patient safety by encouraging the abandonment of validated oncology standards of care.

### Pain management, analgesia, and recovery

Chronic pain affects approximately one in four adults globally and is one of the major reasons why people turn to the CBD market (McDonagh et al. 2022). However, the clinical agreement on the efficacy of CBD for pain remains highly debatable and heavily dependent on the specific formulation and route of administration. Additionally, the clinical efficacy of cannabinoid interventions requires a strict structural separation between pure CBD isolates and multi-cannabinoid combination formulations, as their therapeutic paths are fundamentally distinct.

#### Purified and synthetic CBD isolates

According to the 2025 update of the Living Systematic Review on Cannabis for Chronic Pain, evidence suggested that pure CBD isolates may not be associated with improved systemic pain outcomes compared to placebos (Chou et al. 2026). Another review, by Rojas-Valverde et all. concluded that synthetic or purified CBD alone was not associated with decreased pain intensity versus placebo, with the pooled mean difference actually favoring placebo in some instances (Rojas-Valverde et al. 2023).

Despite the poor systemic efficacy of oral CBD for pain, targeted topical delivery remains a strong market and clinical segment. Transdermal gels applied three times daily have successfully improved grip strength and reduced pain in patients with hand osteoarthritis (Cásedas et al. 2024). Furthermore, a survey by Dahlgren et all. indicates that CBD suppositories are associated with reductions in menstrual and pelvic pain symptoms, pointing to a potential new localized treatment approach (Dahlgren et al. 2024).

In the sport sector, CBD was removed from the WADA prohibited list in 2018, leading to widespread adoption among professional athletes (Karam et al. 2025). A survey of Canadian athletes found that 38% had used CBD, with 93% reporting improved sleep and 77% reporting reduced musculoskeletal pain (Karam et al. 2025). However, physiological intervention trials in laboratory settings where delayed-onset muscle soreness was induced found no significant improvements in non-invasive markers of muscle damage or peak limb torque compared to a placebo (Karam et al. 2025). It has been hypothesized that the perceived benefits are heavily influenced by psychological expectancies and the placebo effect, though the mild anxiolytic properties may indirectly support recovery by facilitating a better sleep cycle.

#### Combined THC:CBD formulations and whole-plant extracts

According to the 2025 update of the Living Systematic Review on Cannabis for Chronic Pain, treatments yield highly different results based on the THC-to-CBD ratio (Chou et al. 2026). Products containing equal ratios (such as Nabiximols) are associated with small improvements in pain severity, supported by moderate strength evidence (Chou et al. 2026).

This clinical variance highlights the functional contribution of THC in multi-cannabinoid delivery platforms. While pure, isolated CBD lacks direct binding affinity for central and peripheral CB1 receptors responsible for ascending pain pathway inhibition, the co-presence of THC actively triggers central pain-modulatory mechanisms. Within balanced 1:1 formulations, CBD functions primarily as a negative allosteric modulator, dampening the CB1-mediated psychotropic and tachycardic side effects of THC, which allow patients to tolerate therapeutic analgesic thresholds that CBD isolates cannot achieve.

### Metabolic syndrome, obesity, and dermatology

A highly promising area of research involves the potential role of CBD in addressing metabolic syndrome and obesity (Botticelli et al. 2025). With obesity affecting over 890 million adults as of 2022, identifying novel metabolic modulators is a critical priority.

The translational applicability of CBD within metabolic syndrome, obesity, and inflammatory dermatosis remains greatly restricted by a heavy reliance on preclinical models. Observations regarding white adipose tissue browning, fasting serum glucose drops, and the reversal of non-alcoholic fatty liver disease (NAFLD) were achieved exclusively in rodent models utilizing substantial dose limits ranging from 25 to 50 mg/kg. These doses do not reflect human physiology, as scaling such quantities to a standard 70 kg adult would necessitate extreme daily intakes that trigger severe hepatotoxicity. In a similar manner, dermatological interventions for acne, psoriasis, and wound healing rely primarily on localized in vitro sebocyte cultures and unblinded topical series. Until large-scale, human randomized controlled trials are completed, these metabolic and cosmetic results cannot be assumed to possess direct clinical applicability. However, the preclinical findings are worth mentioning and can be a real direction of new applications for CBD.

#### Adipose tissue browning and Non-Alcoholic Fatty Liver Disease (NAFLD)

Recent preclinical trials utilizing murine models have demonstrated that CBD supplementation can significantly alleviate metabolic dysfunction (Bravo Iniguez et al. 2025). At doses of 25 to 50 mg/kg, CBD reduced overall weight gain and lowered fasting serum glucose (Bravo Iniguez et al. 2025). The molecular mechanisms involve the browning of white adipose tissue by upregulating Prdm16 and promoting uncoupling protein 1 (UCP1), which enhances thermogenesis and energy expenditure. Further studies found that CBD and CBG may help reverse NAFLD by decreasing hepatic lipid accumulation and sensitizing adipose tissue to insulin (Wiciński et al. 2023). Human trials combining CBD with GLP-1 receptor agonists are actively investigating synergistic potential for Type-2 diabetes control (Wiciński et al. 2023; F02124 - Weightloss and Diabetes Study 2026).

#### Inflammatory dermatosis and wound healing

The human skin possesses an extensive endocannabinoid system, making CBD an attractive compound for dermatology (Rusu et al. 2025). Recent systematic reviews revealed that topical CBD produces a modest but significant reduction in pruritus (severe itching) through neuroimmune modulation (Rusu et al. 2025; Sermsaksasithorn et al. 2025). The conditions and CBD use are summarized in the table below (Table 6).

**Table 6:** CBD use for dermatological conditions

Dermatological Condition	Mechanism of CBD Action	Clinical Potential	Ref
Acne Vulgaris	Regulation of sebum production	Demonstrates capacity to manage sebum production and localized inflammation	Rusu et al. 2025)
Psoriasis	Reduction of keratinocyte hyperproliferation	Beneficial for localized scalp and skin plaques	Rusu et al. 2025)
Pyoderma Gangrenosum	Acceleration of wound healing	Shows ability to treat deep skin ulcers and epidermolysis bullosa	Rusu et al. 2025)
Atopic Dermatitis	Neuroimmune modulation	Modest reduction in itching, but RCTs show mixed results over standard vehicles	Sermsaksasithorn et al. 2025)

### Safety profile

A persistent misconception is that CBD is a universally safe, biologically inert supplement. While it exhibits a favorable safety profile compared to opioids, it exerts profound effects on hepatic metabolism and carries distinct toxicological risks.

#### Hepatic metabolism and CYP450 inhibition

CBD acts as a potent competitive inhibitor of several key CYP isozymes, including CYP3A4, CYP2C19, CYP2C9, and CYP2D6 (Smith et al. 2022). These enzymes metabolize over 60% of pharmaceuticals (Smith et al. 2022). This inhibition causes co-administered drugs to accumulate to potentially toxic levels (Geneau et al. 2025). The interactions between CBD and other known pharmaceuticals are summarized in Table 7.

**Table 7:** CBD drug-drug interaction

Medication Class	Mechanism of CBD Interaction	Consequence	Ref
Anticoagulants (Warfarin)	Inhibition of CYP2C9	Drastic increase in INR; severe risk of spontaneous hemorrhage	Smith et al. 2022)
Anticonvulsants (Clobazam)	Inhibition of CYP2C19	Increases plasma concentration threefold; extreme sedation and respiratory depression	Chen et al. 2024)
Immunosuppressants	Competitive inhibition of CYP3A4	Dangerous accumulation of Tacrolimus or Sirolimus; risk of organ damage	Nanchani et al. 2024)
SSRIs/Antidepressants	Inhibition of CYP2D6/3A4	Increased risk of anticholinergic syndrome and profound drowsiness	Nanchani et al. 2024)

#### Hepatotoxicity and adverse events

In healthy adults, high concentration of CBD use is generally associated with an increased risk of diarrhea (Relative Risk = 5.85) (Sawaira et al. 2026). However, dose-dependent hepatotoxicity remains the primary clinical concern (Chen et al. 2024). Safety data from Epidiolex trials and healthy adult studies demonstrate that patients receiving moderate to high doses (≥ 10–25 mg/kg/day or > 300 mg/day) frequently exhibit elevations in liver enzymes (ALT and AST) exceeding three times the upper limit of normal (Chen et al. 2024). This drug-induced liver injury requires strict monitoring, especially when high-dose CBD is combined with other hepatically metabolized medications (Chen et al. 2024).

To ensure patient safety and prevent drug-induced liver injury, clinicians must move away from treating CBD as an unregulated supplement and instead apply strict risk stratification parameters. Based on safety information from the clinical trial datasets, drug-drug interactions and hepatotoxic enzyme spikes follow explicit, predictable dose-threshold steps (Table 8).

**Table 8:** Clinical risk for CBD administration

Risk	Patient Baseline Profile	Administered Dose Thresholds	Monitoring Guidelines	Ref
Low Risk	Healthy adults; normal baseline liver function; zero concurrent prescriptions	< 5 mg/kg/day (or < 150 mg/day)	Standard over-the-counter threshold. No baseline lab tracking required; monitor for mild gastrointestinal changes	Nanchani et al. 2024)
Moderate Risk	Patients taking secondary non-CYP metabolized drugs; older adults over 65	5–10 mg/kg/day (or 150–300 mg/day)	Obtain baseline liver function tests (LFTs). Repeat serum ALT/AST draws at 1 month and 3 months; monitor for signs of persistent drowsiness	Chen et al. 2024)
High Risk	Patients taking confirmed narrow therapeutic index CYP3A4/2C19 targets (such as Clobazam, Warfarin, Tacrolimus)	≥ 10–25 mg/kg/day (or > 300 mg/day)	Strict Medical Mandate; compulsory baseline LFT tracking before initiation. Repeat LFT serum audits bi-weekly for the first month, then monthly thereafter. Preemptively lower baseline doses of co-administered anticonvulsants or anticoagulants by 25–50% to mitigate sudden systemic toxicity spikes	Smith et al. 2022; Chen et al. 2024; Nanchani et al. 2024)

### Misconceptions and regulatory changes

The rapid commercialization of CBD has promoted a consumer market characterized by unverified health claims that pose risks to public health.

#### The inter-entourage effect

**Fig. 4:**
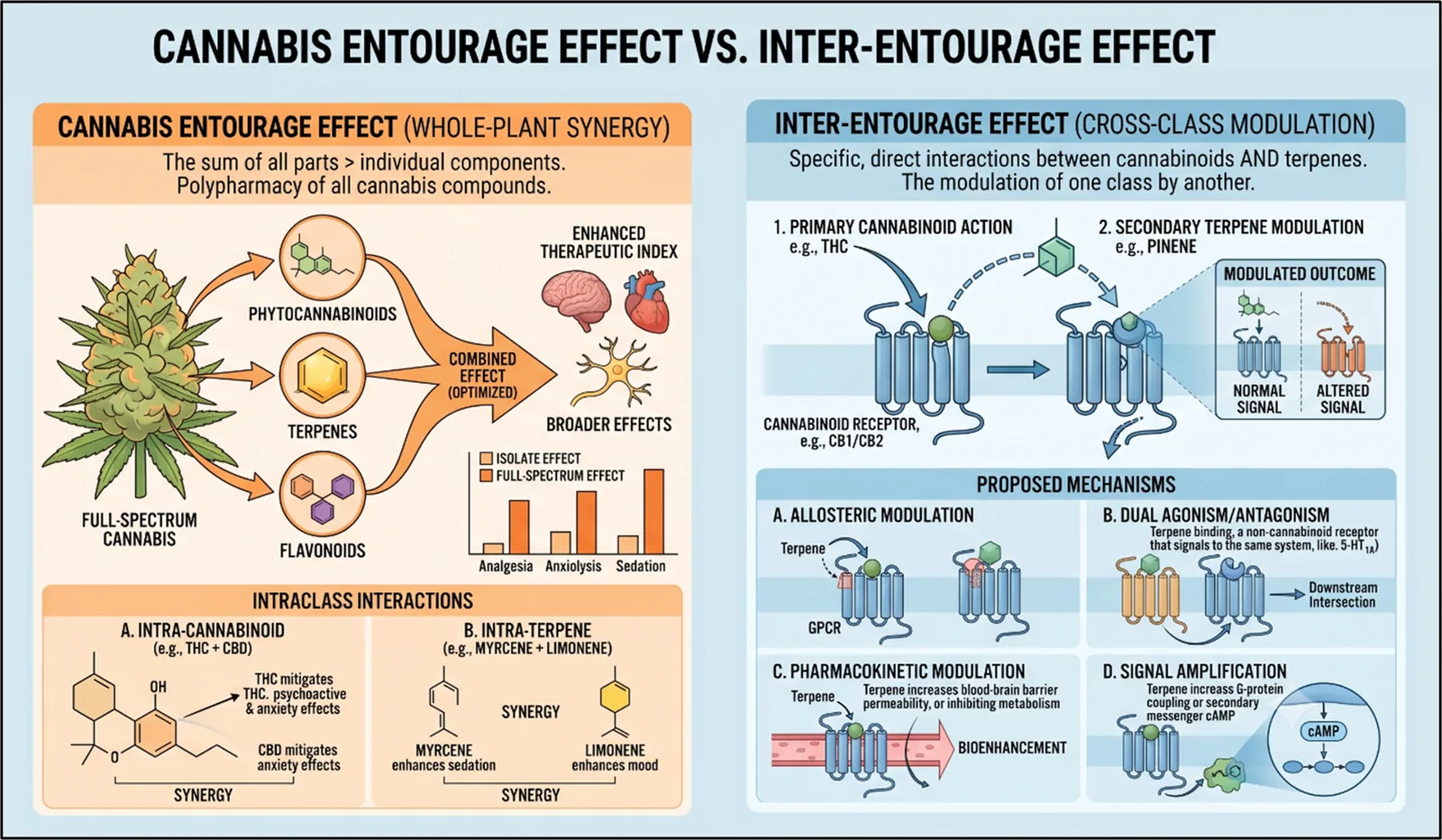
Entourage effect vs inter-entourage effect. The left panel illustrates whole-plant synergy achieved by intraclass polypharmaceutical interactions among cannabinoids, terpenes, and flavonoids to optimize the therapeutic index over single-molecule isolates. This features (**A**) intra-cannabinoid synergy (CBD mitigating THC psychomimetic effects) and (**B**) intra-terpene synergy (myrcene and limonene together modulating sedation). On the contrary, the right panel details the cross-class inter-entourage effect, where secondary terpenes alter primary cannabinoid signal transduction at the receptor interface. This cross-class modulation is hypothesized to occur via four primary molecular mechanisms: (**A**) allosteric modulation of G-protein coupled receptor (GPCR) binding affinity or efficacy; (**B**) dual agonism/antagonism via heterologous receptors (5-HT_1A_) resulting in overlapping intracellular cascades; (**C**) pharmacokinetic modulation enhancing bioavailability through increased membrane fluidity, blood–brain barrier permeability, or metabolic inhibition; (**D**) intracellular signal amplification upregulating G-protein coupling or secondary messengers like cyclic adenosine monophosphate (cAMP)

A persistent marketing claim is that the inter-entourage effect, the hypothesis that minor cannabinoids and terpenes (such as myrcene and pinene) act synergistically with CBD to enhance its efficacy (Russo 2011). Some brands utilize this to justify high pricing for full-spectrum products. However, a comprehensive PRISMA systematic review found strong scientific evidence that is not supporting this claim in clinical settings (André et al. 2024). Topical myrcene combined with CBD showed no anti-inflammatory superiority over isolate, and terpenes such as terpinolene showed no empirical support for mood enhancement in cellular models. The scientific community views the entourage effect largely as a marketing theory, as the low oral bioavailability of ingested terpenes makes systemic neurochemical modulation pharmacokinetically improbable (André et al. 2024). However, a critical distinction exists between intraclass interactions, the established synergistic modulation between cannabinoids, such as the capacity of CBD to mitigate THC-induced psychoactivity, and the hypothesized inter-class inter-entourage effect (Chen et al. 2024). While evidence for the former is strong, the latter remains insufficiently clarified. Initial investigations attempting to quantify synergy between cannabinoids and specific terpenes have frequently failed to report statistically significant advantages over isolated cannabinoid controls (Nanchani et al. 2024). However, these null findings should not be interpreted as a definitive rejection of the hypothesis. It is plausible that early studies lacked the required methodological sensitivity or statistical power to detect complex pharmacological synergy, particularly given the challenges of systemic terpene bioavailability. Therefore, the inter-entourage effect remains a valid area of investigation that necessitates more advanced, high-resolution experimental designs to determine whether meaningful physiological interactions exist beyond the current speculative marketing narratives. The difference between entourage effect (between cannabinoids) and inter-entourage effect (class of compounds from cannabis) is shown in Fig. 4.

#### FDA warnings and unverified claims

Between 2015 and 2025, the U.S. Food and Drug Administration (FDA) issued dozens of warning letters to companies promoting CBD to treat over 125 unique conditions (Wagoner et al. 2015). Unsupported claims included asserting that CBD cures or mitigates Alzheimer's (59% of accused companies), treats schizophrenia (53.8%), and induces cancer cell apoptosis (43.6%) (Wagoner et al. 2015). Regulators emphasize that these practices encourage patients to abandon evidence-based pharmaceutical care in favor of untested extracts that may contain undisclosed heavy metals or synthetic cannabinoids (Wagoner et al. 2015; US FDA 2026).

### Global regulatory landscape

The regulatory control of CBD is currently characterized by policy changes and the reduction of consumer access in response to emerging toxicological data. Frequent changes in global regulations disrupt the entire CBD field, creating severe challenges for both commercial industries and scientific research. For the market, sudden legal updates and shifting safety thresholds destroy predictability, which can make existing inventories legally unviable overnight and force companies into expensive product reformulations.

Besides economics, this instability creates a major bottleneck for scientific progress. The lack of a unified global framework discourages institutional funding, causes difficulties with compliance for multi-center clinical trials, and restricts the international sourcing of standardized, pharmaceutical-grade ingredients. Ultimately, instead of protecting public health, these unpredictable policies drive consumers toward unregulated gray markets while delaying the clinical development of standardized, evidence-based therapies.

#### Thailand 2025 decriminalization reversal to medical only system

In an unexpected shift, Thailand, which fully decriminalized cannabis in 2022, passed a regulatory reversal in mid-2025 (Thailand Cannabis Regulation 2025; Akif et al. 2026), shown in Table 9. Citing a 3.5-fold increase in poisoning cases and a surge in addiction, recreational cannabis was re-criminalized under the Announcement on Controlled Herbs B.E. 2568 (2025) (Thailand Cannabis Laws 2026).

**Table 9:** Thai legal framework change

Thai Regulatory Change (2025–2026)	Operational and Enforcement Impact	Ref
Mandatory Prescriptions	Consumers must obtain Form Phor Thor 33 from a certified doctor to purchase flowers	Thailand Cannabis Regulation 2025; Amendment to Cannabis Law 2025)
Dispensary-to-Clinic Shift	All retail shops must transition into licensed medical clinics by January 2026	Thailand Cannabis Regulation 2025)
Enforcement	Possession without a prescription incurs fines up to 25,000 THB and imprisonment	Thailand Cannabis Regulation 2025)

Notably, non-psychoactive CBD products with less than 0.2% THC remain legal for over-the-counter retail purchase, provided they meet strict manufacturing standards (US_FDA 2026)

#### The European Union and EFSA Safety thresholds

In February 2026, the European Food Safety Authority (EFSA) established its first provisional safe intake level for CBD as a novel food ingredient (N. F. EFSA Panel on Nutrition et al. 2015; Food 2026). Citing concerns over liver toxicity and developmental risks, the EFSA set the safe daily intake at a remarkably low 0.0275 mg per kg of body weight (N.F. EFSA Panel on Nutrition 2026). For a 70 kg adult, this equates to a maximum daily intake of approximately 2 mg, considerably lower than the 10 mg limit previously suggested by the UK's FSA (N.F. EFSA Panel on Nutrition 2026). This mandate explicitly excludes pregnant women, individuals under 25, and anyone taking concurrent medications (Sha et al. 2026). This ultra conservative threshold has severely disrupted the European edible market, forcing massive reformulations for products that typically dose between 25 and 50 mg (N.F. EFSA Panel on Nutrition 2026).

#### United States Federal policy rescheduling

In the United States, federal policy is focused heavily on resolving the contradictions between state-level cannabis legalization and federal prohibition. Executive and legislative efforts are centered on completing the rulemaking process to move cannabis from Schedule I (substances with no accepted medical use) to Schedule III of the Controlled Substances Act (P. United States 2025). This rescheduling formally acknowledges the medical utility of cannabinoids and significantly eases the bureaucratic constraints on federal clinical research, allowing researchers to more freely study the efficacy and safety profiles of CBD without the limitations of Schedule I registration.

### Market dynamics, artificial intelligence, and personalization

Despite regulatory restrictions, the global CBD market was valued between $10.68 billion and $23.94 billion in 2025, with North America capturing over 85% of global revenue (Tomazic 2026). Consumer behavior has changed and successful commercial entities are pivoting towards improved outcome, marketing targeted formulations for sleep applications or support rather than generic CBD (Tomazic 2026).

The rapid inclusion of artificial intelligence (AI) and clinical pharmacogenomics marks an important step away from the old trial-and-error cannabinoid dosing, introducing an analytical solution to the CBD highly variable bioavailability profiles (Tomazic 2026; Ghaffar 2025). Rather than functioning as a passive calculation instrument, modern AI platforms utilize machine learning architectures to solve individual patient metabolic risks by cross-analyzing known pharmacogenomic polymorphisms (Ghaffar 2025; Pam Chmiel 2026). Specifically, single nucleotide polymorphisms within the CYP2C19 (*2 and *3 loss-of-function alleles) and CYP2C9 gene lines predict whether a patient is classified as a poor, intermediate, or ultra-rapid cannabinoid metabolizer (Cásedas et al. 2024; Pam Chmiel 2026). AI-driven clinical dashboards process these specific genetic baselines alongside real-world electronic health records to dynamically predict hepatic clearance kinetics before the first dose is ever administered (Ghaffar 2025; Pam Chmiel 2026). By establishing closed-loop biometric tracking networks, where real-time consumer smart-wearable data tracking heart-rate variability and sleep profiling feed back into the AI model, the algorithm actively scales daily dosing limits (Tomazic 2026; Pam Chmiel 2026). This analytical loop maintains systemic CBD levels within a fitted therapeutic window, maximizing target receptor occupancy while successfully preventing the cannabinoids levels from crossing into the high-dose thresholds known to trigger hepatic enzyme elevation (Cásedas et al. 2024; Geneau et al. 2025; Pam Chmiel 2026; Lakhan et al. 2026). This transition changes AI from a descriptive marketing concept into an actionable system that improves clinical safety dangers connected to commercial cannabinoid variability (Ghaffar 2025; Pam Chmiel 2026).

Besides personalizing patient care, machine learning systems are guiding a major shift in cannabinoid drug discovery through in silico screening and the computational modeling of the entourage effect (Almobaideen et al. 2025; Lavecchia 2015; Atanasov et al. 2021). Given that the cannabis plant contains over 100 minor cannabinoids and hundreds of volatile terpenes, testing every potential combination using traditional in vitro or in vivo assays presents a logistical and financial holdup (Ren et al. 2021; Almobaideen et al. 2025; Lavecchia 2015; Atanasov et al. 2021). AI platforms overcome this problem by employing deep learning neural networks and high-throughput quantitative structure–activity relationship (QSAR) models to run massive structural docking simulations against targeted human receptor topologies, including the CB1 allosteric pocket, 5-HT1A, and TRPV1 channels (Behl et al. 2022; Reggio 2010). Significantly, these systems map complex network pharmacology to predict how non-cannabinoid constituents, such as the dietary sesquiterpene beta-caryophyllene or monoterpenes, such as myrcene and limonene, alter the binding kinetics, membrane permeability, or downstream intracellular signaling cascades of primary cannabinoids (Cásedas et al. 2024; Taming 2011). By calculating multi-ligand synergistic profiles, AI transitions the entourage effect from a speculative botanical concept into a quantifiable molecular pattern (Ghaffar 2025; Taming 2011). This is important to design optimized, multi-target chemical ratios personalized for complex pathologies such as neuroinflammation or refractory pain prior to initiating physical laboratory validation, significantly accelerating the translational drug process (Almobaideen et al. 2025; Lavecchia 2015; Taming 2011).

### Future research directions and priorities

As clinical demand increases, the scientific community must solve significant methodological gaps characterized by small sample sizes and unstandardized extracts (Singh et al. 2025), as shown in Table 10.

**Table 10:** Future directions for CBD studies

Research Priority	Objective	Rationale	Ref
Isolation vs. Spectrum Trials	Large-scale, double-blind RCTs	To definitively prove or disprove the clinical relevance of the entourage effect	Cásedas et al. 2024)
Longitudinal Safety Data	Observational periods > 12 months	To accurately assess chronic liver risk and potential endocrine disruption	Sawaira et al. 2026)
Metabolic Interventions	Translation of successful murine data	To develop FDA-approved treatments for obesity and Type-2 diabetes	Wu et al. 2025)
Precision Delivery	Development of advanced DDS	Bypassing first-pass metabolism via nanocarriers and micro-needle patches	Afonso et al. 2026)

While synthesizing available evidence, it is important to acknowledge that the conclusions are fundamentally shaped by the methodological gaps prevalent in the CBD research landscape, such as the predominance of small sample sizes, heterogeneity in study designs, and the use of unstandardized extracts. These factors collectively limit the certainty of some findings presented herein and make it difficult to extrapolate the results.

## Conclusions

Cannabidiol currently occupies a changing point between its status as a popular consumer wellness supplement and its legitimate potential as a targeted pharmacological agent. The empirical clinical evidence strongly and definitively supports the efficacy of highly purified CBD in managing refractory pediatric seizure disorders and addressing specific symptom distress in oncology palliative care. Within the specific domain of oncology, the boundary between exploratory science and clinical results must be explicitly maintained to protect public health. While in vitro and murine models consistently demonstrate promising localized anti-tumor mechanisms, such as apoptosis induction, cell cycle arrest, and the suppression of metastatic progression, these findings remain strictly preclinical. Currently, there is no high-level human clinical evidence supporting the use of purified or full-spectrum CBD as a primary, curative anti-cancer agent. Instead, its validated clinical utility in oncology is entirely restricted to palliative care, specifically focusing on the management of chemotherapy-induced side effects and overall symptom distress. On the other hand, localized topical formulations show significant promise in pain management and dermatology. However, the use of systemic, over-the-counter CBD for severe psychiatric disorders, muscle repair, or as a primary anti-tumor agent remains unsupported by rigorous, placebo-controlled human trials. As the physiological dose-dependent hepatotoxicity and severe CYP450-mediated drug-drug interactions become more researched, the medical community must actively warn about the claims propagated by the commercial market about CBD. The restrictive regulatory changes seen in Thailand and the European Union in 2025 and 2026 reflect a transition towards a controlled, science-based structure. Arguably, the true potential of CBD will be unlocked not through generic wellness supplements, but through the strategic integration of AI-driven personalized genomic dosing and advanced nanocarrier delivery systems, which were critically analyzed as key solutions to its inherent bioavailability challenges. While we adhered to evidence-based principles, the intrinsic heterogeneity of the primary studies and the rapid pace of research in the CBD field mean that some interpretations are subject to ongoing re-evaluation. Acknowledging these limitations is important for safely and effectively realizing the diverse therapeutic promises of CBD in modern healthcare.

## Supplementary Information

Supplementary Material 1.

## Data Availability

No datasets were generated or analysed during the current study.
