# Supplementary material for: The therapeutic landscape of cannabidiol: a systematic review of molecular mechanisms, clinical efficacy in oncology and neurology, and regulatory changes
Source: J Cannabis Res. 2026 Aug 6;8:118. doi: 10.1186/s42238-026-00482-0 (PMC13729537; doi:10.1186/s42238-026-00482-0)
Supplement: Supplementary file 1 [file 42238_2026_482_MOESM1_ESM.docx]

Supplementary material

**Table S1. Summary of CBD studies in cancer cell lines**

| Year | Cell/Cancer type | Cell line | Objective | Treatment and concentration | Conclusion | MoA** | Ref. |
| --- | --- | --- | --- | --- | --- | --- | --- |
| 2006* | breast cancer | MDA-MB-231 | The main objective was to identify natural cannabinoids with anti-tumor activities similar to or better than THC, but without psychotropic effects | CBD 10 μM;  CBG, CBC, CBD-A, THC-A (1 to 25 μM) | CBD was found to be the most potent inhibitor of cancer cell growth among the tested compounds, with significantly lower potency in non-cancer cells. CBD and CBD-rich extracts showed promising anti-tumor and anti-metastatic effects in vivo | The study found that CBD's anti-cancer effects involve multiple mechanisms: induction of apoptosis and/or cell cycle arrest, depending on the cell type, activation of CB2 and TRPV1 receptors in some cell lines (e.g., MDA-MB-231), elevation of intracellular Ca2+ levels, generation of reactive oxygen species (ROS), and induction of apoptosis via caspase-3 activation in some cell lines | [1] |
|  | glioma | C6 |  |  |  |  |  |
|  | prostate cancer | DU-145 |  |  |  |  |  |
|  | colorectal cancer | CaCo-2 |  |  |  |  |  |
|  | gastric cancer | AGS |  |  |  |  |  |
|  | thyroid cancer | KiMol |  |  |  |  |  |
|  | leukemia | RBL-2H3 |  |  |  |  |  |
| 2012* | Prostate carcinoma | LNCaP, 22RV1, DU-145, and PC-3 | To investigate the anti-cancer potential of non-THC cannabinoids and corresponding botanical drug substances (BDS) in both androgen receptor (AR)-expressing and non-AR-expressing prostate carcinoma cells (PCCs) | CBD 10 μM;  other cannabinoids and BDS | Non-THC cannabinoids, particularly CBD, inhibit prostate carcinoma cell viability, with CBD showing pro-apoptotic effects. CBD-BDS reduced tumor size in xenografts from LNCaP cells and enhanced the anti-cancer effects of bicalutamide | CBD induced apoptosis through multiple pathways, including: elevation of intracellular Ca^2+^ and reactive oxygen species (ROS) production, up-regulation of PUMA and CHOP expression, activation of caspase 3/7, down-regulation of androgen receptor (AR) mRNA in AR-expressing cells, reduction of TRPM8 mRNA levels, possible involvement of GPER (G-protein-coupled estrogen receptor | [2] |
| 2013* | Leukaemia | CEM (acute lymphocytic leukaemia) and HL60 (promyelocytic leukaemia) | To explore the cytotoxic effect of prominent cannabinoids on two leukaemia cell lines, with a particular interest in examining the impact of drug-scheduling in determining activity | CBD (10 μM);  CBDA (20 μM);  CBG (10 μM);  CBGA (40 μM);  CBGV (20 μM);  CBGVA (40 μM); | The study supports the potential role of cannabinoids in cancer treatment, both as single agents and in combination. It highlights the importance of treatment schedules, particularly the inclusion of a recovery phase, which can enhance cytotoxicity | Mechanisms of action of CBD include: cytostatic effect, causing cell cycle arrest, increases p21WAF1 levels, independent of p53, alters pERK levels, does not directly damage DNA, can induce cytotoxicity when followed by a recovery phase | [3] |
| 2014 | colon adenocarcinoma | human epithelial colon adenocarcinoma cell lines HCT 116 and DLD-1, healthy colonic epithelial cells (HCEC) | Inhibition of colon carcinogenesis by CBD and CBD BDS (botanical drug substance) | CBD/CBD BDS at 0.3-5 µM for 24 hours | CBD and CBD BDS exhibited significant inhibitory effects on cell proliferation in colorectal cancer cell lines (assessed by the MTT assay) | CBD via CB1, CBD BDS via CB1 and CB2 | [4] |
| 2014 | colorectal cancer | Human Colorectal cancer, Caco-2 | Modulation of cell lines through physiological intestinal oxygen to increase CBD sensitivity | CBD 0.1–10 µM | PhysO2 cells are significantly more sensitive to antiproliferative effects of CBD than AtmosO2 | CBD deactivated the mTOR/S6 signaling pathway, which is involved in regulating cell size | [5] |
| 2014 | glioma | T98G (GBM), U87MG (glioblastoma astrocytoma) | To assess the efficacy of CBD and THC in combination with radiotherapy for glioma treatment | CBD and THC in pure (P) and botanical drug substance (BDS) forms (0.1-100 μmol/L); Irradiation (0 to 20 Gy); | The combination of CBD, THC, and radiotherapy significantly inhibited tumor progression in an orthotopic syngeneic model for glioma | The treatment enhanced radiosensitivity of glioma cells, delayed DNA damage repair, induced autophagy and apoptosis, inhibited tumor angiogenesis, alterations in intracellular signaling pathways (MAPK, PI3-kinase/AKT) | [6] |
| 2015 | glioblastoma | Human Glioblastoma, U878MG, U373MG | Induction of ROS-mediated therapeutic response | CBD 5–40 µM | Antiproliferative effects correlated to induction of apoptosis | CBD was found to inhibit GSC viability at concentrations of 2-3.5 μM by increasing ROS | [7] |
|  | primary glioma stem | Human Glioblastoma,  U251, 3832, 387  Primary glioma stem  cells (GSC) lines |  | CBD 0–5 µM | Activation of p-p38 pathway, downregulation of key stem cell regulators; Sox2, Id1 and p-STAT3 |  |  |
| 2016 | macrophage | LPS-stimulated murine macrophage RAW cells | Evaluate the protective effects of a combination of low doses of CBD (5 μm) and moringin (5 μm) | CBD (5 μM) + moringin (5 μM) | CBD-moringin cocktail reduced LPS-induced morphological changes in macrophages. | CBD appears to act primarily through TRPV1 receptors | [8] |
| 2016 | neuroblastoma | Human Neuroblastoma, SK-N-SH, IMR-32, NUB-6 and LAN-1 | In vitro and in vivo efficacy of CBD | CBD 0–50 µg/mL | Cell viability reduction and apoptosis | CBD induced apoptosis in NBL cells, as evidenced by morphology changes, sub-G1 cell accumulation, annexin V assay results, and increased cleaved caspase 3 expression | [9] |
| 2016 | Cervical cancer | SiHa, HeLa, and ME-180 | To compare the anti-proliferative effects of crude extract of Cannabis sativa and its main compound cannabidiol on different cervical cancer cell lines | *Cannabis sativa* extracts: 50, 100, and 150 μg/ml; CBD: concentrations varied by cell line - SiHa and HeLa (3.2 μg\ml), ME-180 (1.5 μg\ml) | Cannabidiol rather than *Cannabis sativa* crude extracts prevent cell growth and induce cell death in cervical cancer cell lines | CBD inhibited cell proliferation, induced apoptosis without cell cycle arrest, ATP depletion in cancer cells, up-regulation of pro-apoptotic proteins (p53, Bax), down-regulation of anti-apoptotic proteins (RBBP6, Bcl-2), activation of caspase-9 and caspase-3, and apoptosis via the mitochondrial pathway | [10] |
| 2017 | leukemia | Human T acute lymph oblastic leukaemia, Jurkat | Cannabidiol influence on leukemic cell size | CBD 0.01–10 µM | Reduction in cell viability and cell cycle changes | CBD deactivated the mTOR pathway, particularly reducing phosphorylation of ribosomal protein S6 | [11] |
| 2018 | pancreatic cancer | AsPC-I, HPFA-II, PANC-I, BxPC-3 | GPR55 signalling and proliferation of pancreatic cancer cells | CBD 0–10 µM | Antiproliferative effects via GPR55 | CBD inhibited GPR55, which leads to reduced cancer cell proliferation, cell cycle progression, and activation of MAPK signaling | [12] |
| 2018 | Colorectal cancer | SW480, SW620, HT-29, DLD-1, HCT116, LS174, and RKO | To identify compounds from a synthetic cannabinoid library that universally reduces cell viability of seven distinct CRC cell lines | 370 synthetic cannabinoids were screened at a concentration of 10 μM for 48 hours | The study identified 10 synthetic cannabinoids demonstrating reproducible activity against human CRC cells, with seven compounds reducing viability only in CRC cells. These compounds fall into three families of molecules: CP 55,940, CP 47,497, and PTI | The study suggests that CBD might be able to reduce the activity of the Wnt/β-catenin pathway before the translocation of β-catenin into the nucleus. However, the exact mechanism was not fully elucidated. The study also noted that CBD, unlike THC, is an antagonist of GPR55, which may contribute to its effects on CRC cells | [13] |
| 2018 | Endometrial cancer | Ishikawa (Type 1 EC), Hec50co (Type 2 EC), HFF-1 (non-tumoral cell line) | To characterize cannabinoid elements and evaluate the effect of cannabinoids on endometrial cancer cell viability | Endocannabinoids (AEA and 2-AG): 0.01-25 μM;  Phytocannabinoids (CBD and THC): 0.01-25 μM; | Cannabinoids, particularly AEA and CBD, modulate endometrial cancer cell death. Selective targeting of TRPV1 by AEA, CBD, or other stable analogues may be a promising research area for treating estrogen-dependent endometrial carcinoma. CBD and CBD-rich extracts show potential for treating endometrial cancer, especially in cases non-responsive to common therapies | Reduction of cell viability in both Ishikawa and Hec50co cells. In Ishikawa cells: induction of apoptosis through TRPV1 activation, increase in caspase-3/7 activities, increase in cleaved PARP, generation of reactive oxygen species (ROS), activation of TRPV1 receptor leading to increased intracellular calcium levels. In Hec50co cells: cytotoxic effect independent of cannabinoid or TRPV1 receptors. Potential involvement in ER stress, as indicated by increased CHOP expression in both cell lines | [14] |
| 2019 | lymphoblastic leukemia | Primarily Jurkat cells, but also MOLT-3, CEM, K562, Reh, and RS4;11 | To investigate the anticancer properties and molecular targets of CBD in T lineage in acute lymphoblastic leukemia cells | CBD 10-100 µM | CBD directly targets mitochondria in T-ALL cells, altering their capacity to handle Ca^2+^, which affects multiple cellular functions and can lead to cell death | CBD causes mitochondrial Ca^2+^ overload, induces mitochondrial permeability transition pore (mPTP) formation, leads to loss of mitochondrial membrane potential, causes cytochrome C release, triggers reactive oxygen species (ROS) production, activates caspase-dependent apoptosis, and induces autophagy at sublethal concentrations | [15] |
| 2020 | colon cancer | HT-29, SW480 | To assess the viability and proliferation of cancer cells treated with cannabidiol in presence of a serum concentration that commonly sustains cell growth (10% serum) | CBD up to 10 µM  Paclitaxel at 10 nM | The effect of cannabidiol is cell context-dependent, being modulated by the presence of growth factors. CBD's impact varies depending on serum concentration and cell line | CBD can impair DNA replication in some cell lines (SW480 and AGS) even in 10% serum conditions.  At high concentrations (10 µM), CBD can induce mitochondrial stress and cell death in low serum conditions, while low concentrations of CBD may increase mitochondrial Ca^2+^ and metabolism, while high concentrations can lead to excessive mitochondrial Ca^2+^, dysfunction, and cell death | [16] |
|  | gastric cancer | AGS |  | CBD up to 10 µM  Paclitaxel at 2 nM |  |  |  |
| 2020 | cutaneous lymphoma | My-La (MF) and HuT-78 (Sézary) cell lines, and peripheral blood lymphocytes from Sézary patients (SPBL) | To identify active compounds from whole cannabis extracts and their synergistic mixtures, and to assess respective cytotoxic activity against CTCL cells | 5 μg/mL of S4 fraction: CBD (98.3%), THC (0.3%), CBG (0.2%), α-bisabolol (0.9%), CBDV (0.09%);  6 μg/mL S5 fraction: CBG (58.8%), CBD (38.2%), THC (0.7%), CBC (0.4%);  Synergistic combination of S4 (5 μg/mL) + S5 (6 μg/mL); | The defined S4+S5 formulation of synergistic phytocannabinoids induced cell cycle arrest and cell apoptosis, and affected multiple biological pathways associated with cancer. This combination shows potential for the development of new cannabis-based therapies for CTCL, pending human clinical trials | Induction of apoptosis in CTCL cell lines and SPBL, cell cycle arrest (G2-M phase in My-La, S phase in HuT-78), upregulation of stress-related genes ATF4 and TRIB3, modulation of genes involved in CTCL progression (e.g., NFKBIZ, RRM2, SATB1, PIK3R3), affects multiple biological pathways, including PI3K-AKT pathway; the activity may not be primarily mediated through CB1 or CB2 receptors | [17] |
| 2020 | breast cancer | Human Breast Cancer, MDA-MB-231, MCF-7 | CBD loaded microparticles as a potential formulation to improve paclitaxel and doxorubicin-based chemotherapy | CBD 1–50 µM | Co-administration of CBD and paclitaxel or docetaxel showed a synergistic effect | CBD pre-treatment sensitized cancer cells to PTX and DOX, while co-administration showed synergistic effects | [18] |
|  | ovarian cancer | Human ovarian cancer, SKOV-3 |  | CBD 10–50 µM | Inhibition of cell proliferation |  |  |
| 2020 | colorectal cancer  melanoma  glioblastoma | HCT116, SW480 (colorectal cancer), 1205Lu, A375M (melanoma), T98G, U87MG (glioblastoma) | To investigate the effects of CBD and CBD oil on cancer cell viability and to assess the "entourage effect" | CBD pure 10 μM or CBD from hemp oil 10 μM for 48 hours | CBD reduced cell viability in specific cancer cell lines, indicating that CBD may restrict growth rather than induce apoptosis, and the efficacy was not universally applicable across all tested cell lines. | The mode of action appears to be growth restriction rather than inducing apoptosis | [19] |
| 2021 | breast cancer | MDA-MB-231, Human umbilical cord-derived mesenchymal stem/stromal cells (hUCMSCs) | The characterization and formulation of CBD encapsulated extracellular vesicles (EVs) | CBD (5 mg/kg), CBD EVs (5 mg/kg), and DOX (2 mg/kg) to nude mice | CBD encapsulated in extracellular vesicles enhances the therapeutic efficacy of doxorubicin in breast cancer treatment. | CBD EVs sensitized MDA-MB-231 cells (a TNBC cell line) to doxorubicin | [20] |
| 2021 | glioma | Human - U87MG, U373MG Uppsala; Canine - J3TBG, SDT3G | Investigate CBD cytotoxicity and mechanism of action in glioma cells | CBD isolate and extract (0-20 μg/ml), various inhibitors (zVAD-fmk, necrostatin-1, GSK'872, VX-765, IRTX, DIDS) | CBD induces apoptosis, perturbs mitochondrial function, involves VDAC1 channel, and RIPK3 kinase in glioma cell death | CBD induces apoptosis in glioma cells via dysregulation of calcium homeostasis and mitochondrial activity, particularly through the VDAC1 channel | [21] |
| 2021 | ovarian cancer | SKOV-3, Human brain vascular pericytes, Caco-2 | Compare effects of natural vs synthetic CBD on cancer cell viability, neuroprotection in stroke model, and intestinal barrier function | CBD 10-50 μM (SKOV-3)  CBD 10-100 nM (Pericytes) CBD 10 μM (Caco-2) | CBD exhibits similar anticancer effects regardless of its source, with effective concentrations identified for further pharmacological applications. | Non-receptor-mediated mechanism, possibly involving effects on mitochondrial membrane potential and/or ROS generation | [22] |
| 2021 | breast cancer | MDA-MB-231, MDA-MB-468 | To investigate the anti-cancer potential of CBD and its ability to overcome resistance to chemotherapeutics | CBD (2.5 µM) and Doxorubicin (DOX) (2 mg/kg i.v. Twice a week) | CBD shows anticancer activity against triple-negative breast cancer cells and acts as a potent chemosensitizer for conventional chemotherapeutics. | CBD induces apoptosis through upregulation of GADD45A, phosphorylated p38, and p53 proteins, cause G1 cell cycle arrest and inhibits autophagy by decreasing Beclin1 and ATG-5, -7, and -16 proteins.  CBD also demonstrated anti-migratory effects by decreasing fibronectin, vimentin, and integrins α5, β5, and β1. | [23] |
| 2021 | medulloblastoma, ependymoma | - Medulloblastoma: D283, D425, PER547 - Ependymoma: IC-1425EPN, DKFZ-EP1NS | Investigate the effects of THC and CBD on pediatric brain cancers | THC: 7.5-15 mg/kg; CBD: 30-50 mg/kg; Cyclophosphamide (CPA): 100 mg/kg; Combinations of THC/CBD with CPA | Despite showing cytotoxic activity against medulloblastoma and ependymoma cells in vitro, THC and CBD did not improve survival in mouse models of these pediatric brain cancers, suggesting limited clinical benefit for children with high-risk brain tumors | CBD appeared to induce cell death partly through the production of ROS, induction of autophagy, and cell cycle arrest | [24] |
| 2021 | liver cancer | HepG2 (liver cancer) HeLa (cervical cancer)  A549 (lung cancer) LO2 (normal liver cells) | Develop targeted drug delivery system to Improve solubility and targeting of CBD | AD-CBD (amantadine-conjugated CBD);  BIO-CD (biotin-conjugated cyclodextrin);  AD-CBD/BIO-CD inclusion complex:  -0.15-0.40 mM (for standard curve) - IC50 values in μM range | AD-CBD/BIO-CD complex formed successfully. It improved water solubility of CBD by ~50,000-fold, pH-dependent drug release, enhanced anticancer activity and targeting compared to free CBD. Selective toxicity to cancer cells over normal cells. BIO-CD showed IC50 values >100 μM for all cell lines, and AD-CBD/BIO-CD also showed IC50 >100 μM for LO2 cells. | The BIO-CD targets cancer cells that overexpress biotin receptors. The AD-CBD forms an inclusion complex with BIO-CD, allowing for target delivery and pH-dependent release in the acidic tumor microenvironment. | [25] |
|  | cervical cancer |  |  |  |  |  |  |
|  | lung cancer |  |  |  |  |  |  |
| 2021 | Bladder cancer | T24 and TCCSUP human bladder transitional cell carcinoma cell lines | To investigate the anti-cancer properties of cannabinoids, particularly cannabidiol (CBD) and Δ9-tetrahydrocannabinol (THC), and their potential synergistic effects with gemcitabine, cisplatin, and other cannabinoids in bladder cancer | CBD: IC50 of 5.5 μM in T24 cells and 10.5 μM in TCCSUP cells; THC: IC50 of 8.5 μM in T24 cells and 13.5 μM in TCCSUP cells;  Other cannabinoids (e.g., cannabichromene, cannabivarin) and chemotherapeutic agents (gemcitabine, cisplatin) were also tested at various concentrations (ratio: 1:125) | CBD and THC can reduce human bladder transitional cell carcinoma cell viability and potentially exert synergistic effects when combined with other agents | CBD activates the apoptotic cascade, involving caspase 3 cleavage, reduces invasion in a Matrigel assay, and shows synergistic properties when combined with other cannabinoids or chemotherapeutic agents | [26] |
| 2022 | Colorectal adenocarcinoma | - HT29 (colorectal) - U87MG (glioblastoma) - K562 (leukemia) | To characterize different hemp extracts and assess their antiproliferative activity on various cancer cell lines, focusing on the mechanism of cell death | Raw hemp extracts (R1, R2, R3);  Decarboxylated hemp extracts (D1, D2, D3); Pure CBD:  - 5-100 μg/ml for extracts | The CBD-rich hemp extract (D1) showed potent antiproliferative activity against K562 leukemia cells, inducing apoptosis through caspase activation and mitochondrial disruption, suggesting its potential as a candidate for myeloproliferative disease therapy, either alone or in combination with other anticancer drugs | Apoptotic process attributed to the activation of caspase 3 and 7, cytochrome c release into the cytosol and modulation of the mitochondrial membrane potential | [27] |
|  | Glioblastoma |  |  |  |  |  |  |
|  | Chronic myelogenous leukemia |  |  |  |  |  |  |
| 2022 | Hepatocellular carcinoma | HepG2 and Huh-7 (liver cancer), and - HUVECs (normal cells, used as control) | To investigate the anticancer effects of cannabis-derived extracellular vesicles (CDEVs) on HCC cell lines | High-CBD EVs (H.C-EVs), Low-CBD EVs (L.C-EVs) - 0, 25, 50, 100, 150, and 200 μg/ml | -H.C-EVs significantly reduced viability of HCC cells in a dose-dependent manner, induced G0/G1 cell cycle arrest in HCC cells and triggered apoptosis in HCC cells via mitochondrial-dependent pathway. H.C-EVs showed no significant cytotoxic effect on normal HUVECs cells  while L.C-EVs showed less pronounced effects compared to H.C-EVs. | Cell cycle arrest in the G0/G1 phase and induction of apoptosis via the mitochondrial-dependent pathway.  H.C-EVs treatment led to upregulation of pro-apoptotic genes (BAX, CASP3, CASP8, CASP9) and downregulation of the anti-apoptotic gene Bcl-xl. | [28] |
| 2022 | Epidermoid carcinoma, lung cancer | A431 (EGFR-positive epidermoid carcinoma), A549 (EGFR-positive lung cancer), NIH/3T3 (normal mouse fibroblast) | Assess binding affinity and inhibitory properties of cannabinoids on EGFR-TK; Evaluate anticancer activity | CBD, CBG, CBN up to 10 μM | CBD and CBG showed potent EGFR-TK inhibition, high binding affinity, and significant anticancer activity in EGFR-positive cells. CBD most effective | CBD showed high binding and potent inhibitory activity against EGFR-TK  Molecular docking and dynamics simulations revealed that CBD could stably dock into the ATP-binding site of EGFR-TK through multiple weak interactions | [29] |
| 2022 | Triple-negative breast cancer | MDA-MB-231 | To investigate the effects of CBD on triple-negative breast cancer cells, particularly its antiproliferative effects and interactions with conventional chemotherapy agents | CBD 1-50 μM; Cisplatin (DDP) at 5 μM and 10 μM; IGF-1 5-100 ng/mL; | CBD's effect on MDA-MB-231 cells is strongly associated with its physical state and concentration, which are influenced by serum levels in the culture medium. The study revealed three distinct behaviors at different CBD concentration ranges, and identified an antagonistic effect of CBD on cisplatin treatment. IGF-1 was found to counteract CBD's antiproliferative effect | At intermediate doses (near threshold), CBD induces cell cycle arrest, autophagy, temporary loss of cell adhesion. At high doses, CBD activates bubbling cell death. CBD may interact with IGF-1 receptor, competing with IGF-1 binding | [30] |
| 2023 | Cervical cancer | HeLa (cervical cancer) and WS1 (normal human skin fibroblasts) | To investigate the cytotoxic effects of combinative ZnPcS4 photosensitizer photodynamic therapy (PDT) and CBD on cervical cancer cells | ZnPcS4 PDT: 0.125 μM;  CBD: 0.5 μM;  Laser irradiation: 673 nm wavelength, 10 J/cm² fluency; | The combined treatment approach can possibly induce primary cellular destruction, as well as limit CC metastatic spread | CBD was found to induce apoptosis in CC cells by activating CB1, CB2, and TRPV1 receptors, leading to caspase cascade-mediated programmed cell death | [31] |
| 2023 | Breast cancer | Cancer: MDA-MB-231 (breast), PC-3 (prostate); Normal: MCF-10A (breast), PNT2 (prostate); Other: MSU-1.1 (fibroblasts), HEK-293 (kidney); | Assess and compare the pro-apoptotic potential of CBD-rich Cannabis sativa extracts and purified CBD on cancer cell lines while investigating their potential mechanisms of action | Pure CBD: 0-15 μM; CBD-rich *Cannabis sativa* extracts (B and D) | CBD and CBD-rich *Cannabis sativa* extracts demonstrated selective pro-apoptotic effects on breast and prostate cancer cells, particularly in low-serum conditions, while sparing normal cells, suggesting potential for future preclinical and clinical testing as novel anti-cancer agents | The pro-apoptotic action may involve endoplasmic reticulum stress, as indicated by increased expression of stress-related genes like DDIT3. | [32] |
|  | Prostate cancer |  |  |  |  |  |  |
| 2023 | Bladder cancer (transitional cell carcinoma) | T24, TCCSUP | To evaluate the anticancer effects of cannabinoids alone and in combination with chemotherapy drugs on bladder cancer cell lines, assessing their impact on cell viability, apoptosis, and invasion. | CBD, THC, CBC, CBV (up to 10 μM); Gemcitabine (up to 100 nM);  Cisplatin (up to 100 μM); | Cannabinoids reduce bladder cancer cell viability, induce apoptosis, decrease invasion, and demonstrate potential synergistic effects with chemotherapy drugs, suggesting their possible utility in bladder cancer treatment | CBD induced apoptosis via caspase-3 cleavage and reduced invasion in a Matrigel assay. | [33] |
| 2023 | Colorectal cancer | MC38 (mouse colon cancer cell line) | To elucidate the specific anticancer mechanism of cannabidiol (CBD) using single-cell RNA sequencing (scRNA-seq) and single-cell ATAC sequencing (scATAC-seq) technologies | CBD (5 mg/kg and 10 mg/kg, i.p., once per day); 5-fluorouracil (5-Fu) (25 mg/kg, i.p., once every other day); Anti-PD-1 (200 μg/mouse, i.p., every three days); | CBD inhibits colorectal cancer progression by modulating the tumor microenvironment, specifically by suppressing M2-like macrophages, promoting M1-like macrophages, altering macrophage metabolism through PI3K-Akt signaling inhibition, and enhancing the response to anti-PD-1 immunotherapy. | CBD inhibits colon cancer progression primarily by modulating the tumor microenvironment (TME). CBD was found to suppress M2-like macrophages and promote M1-like macrophages in tumors. CBD altered the metabolic pattern of macrophages by inhibiting the PI3K-Akt signaling pathway | [34] |
| 2024 | hepatocellular carcinoma | human hepatocellular carcinoma-derived cell line HepG2 | Safety profile of cannabidiol (CBD) and its potential therapeutic applications | CBD isolate (CBD CP) and CBD-rich Cannabis sativa L. extract (CBD EX) ranging from 0.05 to 50 μg/ml for cytotoxicity tests; up to 5 μg/ml for genotoxicity tests | Both CBD samples were non-mutagenic in the Ames assay and non-genotoxic at non-cytotoxic concentrations in HepG2 cells | CBD undergoes substantial metabolism, particularly through CYP450 mixed-function oxidases, resulting in various hydroxylated metabolites (7-OH-CBD, direct CBD glucuronide, and 7-COOH-CBD) | [35] |
| 2024 | Chronic myelogenous leukemia | K562 | To identify CBD protein targets and investigate its mechanism of action | CBD: 1 μM, 10 μM, 100 μM for proteomics experiments; 20 μM for cell treatment | CBD interacts with multiple protein targets, including EFTUD2, potentially altering spliceosome functionality | CBD interacts directly with EFTUD2 explaining  CBD's ability to inhibit K562 cell proliferation by inducing apoptosis. | [36] |
| 2024 | Cholangiocarcinoma (CCA), specifically gemcitabine-resistant CCA | - KKU-213BGemR (gemcitabine-resistant CCA cells) - OUMS-24/P6X (non-cancerous immortalized fibroblast cells) | Investigate effects of CBD on gemcitabine-resistant CCA cells in vitro and in vivo and explore mechanism of CBD action against gemcitabine-resistant CCA | CBD: 2.5 to 30 μM | CBD exhibits potent anti-cancer activity against gemcitabine-resistant CCA. CBD induces apoptosis and cell cycle arrest via ER stress-mediated mechanisms. Results warrant clinical exploration of CBD in gemcitabine-resistant CCA patients. | CBD treatment increased expression of ER stress-associated proteins (p-PERK, BiP, ATF4, CHOP) and apoptosis-related proteins (BAX, cytochrome c) | [37] |
| 2024 | Pancreatic ductal adenocarcinoma (PDAC) | Panc03.27, Panc1, HPAF-II, CFPAC-I, IMR-90, murine-3275 | To investigate cannabidiol's (CBD) cytotoxic effects and mechanism of action in PDAC | CBD: 10-40 μM; Gemcitabine: 0.008-0.53 μM; | CBD exhibits dose-dependent cytotoxicity in PDAC cells, potentially through a CerS1-dependent pathway activating ER stress | Upregulation of ceramide synthase 1 (CerS1), which leads to endoplasmic reticulum (ER) stress and activation of the unfolded protein response (UPR) pathway resulting in cell death through the GRP78/ATF4/CHOP arm of the UPR | [38] |

* studies before 2014

**mechanisms of action

**Table S2. Summary of in vivo studies on CBD**

| **Cancer studies** | | | | | | |
| --- | --- | --- | --- | --- | --- | --- |
| **Year** | **Tissue/cancer type** | **Animal model** | **Purpose** | **Treatment used (concentration)** | **Conclusion of study, mode of action** | **Ref.** |
| 2014 | colorectal carcinoma | ICR mice for azoxymethane (AOM)-induced colon cancer model and nude mice for xenograft model of colon cancer in athymic | To investigate the effect of a standardized *Cannabis sativa* extract with high content of cannabidiol (CBD BDS) on colorectal cancer cell proliferation and in experimental models of colon cancer in vivo | CBD BDS 5 mg/kg | CBD BDS attenuates colon carcinogenesis and inhibits colorectal cancer cell proliferation via CB1 and CB2 receptor activation | [4] |
| 2015 | glioblastoma,  glioma stem cells | Female Athymic (nu/nu) mice | The study aimed to investigate mechanisms underlying GSC response and resistance to CBD | CBD 15 mg/kg | CBD exhibits significant antitumor activity against GSCs by increasing ROS. | [7] |
| 2016 | neuroblastoma | Immunodeficient (NOD/SCID)  mice | To investigate the anti-neuroblastoma effect of cannabinoids, specifically THC and CBD | CBD 20 mg/kg | CBD reduced viability of neuroblastoma cell lines in a dose- and time-dependent manner, more effectively than THC, by induced apoptosis via morphology changes, sub-G1 cell accumulation, positive annexin V assay, increased cleaved caspase 3 and reduced invasiveness of neuroblastoma cells by causing G1 phase cell cycle arrest | [9] |
| 2018 | pancreatic ductal adenocarcinoma (PDAC) | Mice with homozygous deletion  of GPR55 created KPCG strain in KPC PDAC mouse model (mutations in KRAS, PanIN, TP53) | To investigate the role of GPR55 in PDAC development and progression, and to evaluate the potential of targeting GPR55 as a therapeutic strategy | CBD 100 mg/kg  or  Gemcitabine (GEM) 100 mg/kg  or  combination of CBD and GEM | Targeting GPR55, particularly in combination with GEM, represents a novel and promising therapeutic strategy for PDAC treatment. | [12] |
| 2024 | Cholangiocarcinoma | Female nude mouse (BALB/CAJcl-Nu/Nu), 6-7 weeks old, injected with KKU-213BGemR cells | Investigate the anti-tumor effect of CBD on gemcitabine-resistant cholangiocarcinoma (CCA) | CBD in sesame oil at 10 mg/kg·Bw  CBD in sesame oil at 40 mg/kg·Bw  Sesame oil (vehicle control) | CBD exhibits potent anti-cancer activity against gemcitabine-resistant CCA via ER stress-mediated mechanisms.  CBD was found to suppress cell proliferation and colony formation, induce apoptosis and cell cycle arrest, increase ROS production, activate ER stress-associated apoptosis proteins, and suppress tumor growth in the xenograft model | [37] |
| 2024 | Glioblastoma | Orthotopic GBM mouse model (C57BL/6 mice) | investigate the effects of inhaled CBD on stress granules (SGs) in GBM tumor cells | Inhaled CBD (8.56 mg/day) | CBD significantly up-regulated stress granules in GBM.  CBD treatment increased the volume of TIAR-1 (a marker for stress granules) and elevated both eIF2α expression and p-eIF2α in CBD treated tissues. | [39] |
| 2024 | Pancreatic ductal adenocarcinoma (PDAC) | To evaluate CBD's antitumor effects alone and in combination with chemotherapy | FVB/NJ female mice, injected with 3275-Luc+ cell xenografts | CBD: 100 mg/kg thrice weekly, gemcitabine: 10 mg/kg twice weekly, and abraxane: 10 mg/kg twice weekly | CBD upregulates CerS1, inducing ER stress and activating the GRP78/ATF4/CHOP pathway, potentially enhancing chemotherapy effects. | [38] |
| **Other studies** | | | | | | |
| 2022 | brain | Adult male Sprague Dawley rats | To evaluate the effects of single and repeated CBD exposure on Brain-Derived Neurotrophic Factor (BDNF) expression and signaling in the cortico-striatal brain network | Single CBD treatment: 5 mg/kg, 15 mg/kg, or 30 mg/kg;  Repeated CBD treatment: 5 mg/kg, 15 mg/kg, or 30 mg/kg for seven consecutive days | A single high dose (30 mg/kg) of CBD upregulated Bdnf exon IV, increased cortical mBDNF and TrkB, and activated the PI3K pathway in the mPFC. Repeated CBD exposure slightly decreased mBDNF, TrkB, and Akt activation in the mPFC, but increased mBDNF in the striatum. This suggests that CBD may target specific neuroplastic processes in the cortico-striatal pathway | [40] |
| 2022 | neuronal health | Caenorhabditis elegans (C. elegans) | To investigate the effects of CBD on lifespan, healthspan, and neuronal aging, and to explore the role of autophagy and SIRT1/sir-2.1 in these processes | CBD at 1 μM | CBD extends lifespan, improves healthspan, induces autophagy, and enhances neuronal health through the SIRT1/sir-2.1-autophagy pathway. CBD's effects on longevity and neuronal aging are dependent on autophagy genes (bec-1, vps-34, and sqst-1) and sir-2.1 (the C. elegans ortholog of SIRT1). | [41] |
| 2023 | liver | Sprague-Dawley rats (male and female, approximately 7–7.5 weeks of age). | To evaluate the genotoxicity and oral repeat-dose toxicity of CBD in rats. | CBD at doses of 0 (vehicle control), 250, 500, or 1000 mg/kg-bw/d once daily for two consecutive days | There was no statistically significant or dose-dependent increase in micronucleus formation in male or female rats at any CBD dose level, indicating no genotoxic potential | [42] |
| 2023 | Several types of tissues and blood | Sprague-Dawley CD IDS rats | To evaluate the effects of CBD isolate on various health parameters through a 14-day repeat dose range-finding study and a 90-day repeated dose toxicity trial. | Control group received 100% MCT (medium-chain triglyceride);  Groups 1 to 3 received 115, 230, and 460 mg/kg bw/d of CBD isolate. | The CBD isolate was well tolerated with no significant dose-related toxic effects up to 230 mg/kg bw/d. Lowest-Observed-Effect-Level (LOEL): The study identified a LOEL of 30 mg/kg bw/d for both males and females.  Using an uncertainty factor of 100, the study suggests that subchronic intake of this specific CBD isolate is safe for reference adult humans (70 kg) at a dose of 161 mg per day. | [43] |

**Table S3. Summary of clinical trials investigating CBD in cancer symptoms management**

| **Cancer type** | **Type of study** | **Location**  **(year)** | **Subjects (n)** | **Objective** | **Treatment used (concentration)** | **Conclusion of study** | **Ref.** |
| --- | --- | --- | --- | --- | --- | --- | --- |
| Advanced cancers | Prospective two-arm open-label pilot trial | Australia  (2020) | 21 (16 CBD, 5 THC) | Assess feasibility of using global symptom burden measures, determine median tolerated doses, document adverse events | CBD: 100 mg/mL oral oily solution, dose range 50-600 mg/day; THC: 10 mg/mL oral oily solution, dose range 2.5-30 mg/day; | CBD was generally well-tolerated at a median dose of 300 mg/day in advanced cancer patients undergoing palliative care, with some patients showing marked improvement in total symptom distress, although further placebo-controlled trials are needed to confirm its efficacy. | [44] |
| Advanced cancers | A phase IIb, randomized, placebo-controlled, dose-escalating, double-blind trial | Australia  (2022) | 144 (72 per arm) | Advanced cancer and substantial symptom distress | Median daily dose of 400 mg of CBD (range, 50-600 mg) | CBD oil did not demonstrate a statistically significant improvement in total symptom distress scores compared to placebo on day 14. | [45] |
| Advanced cancers | Randomized controlled trial (RCT) | Australia  (2023) | 141 participants (71 placebo, 70 CBD) | To assess anti-inflammatory effects of CBD in advanced cancer patients | CBD oil vs placebo, escalating doses (50 to 600 mg/day) | No detectable anti-inflammatory effect of CBD in cancer patients. | [46] |
| Breast cancer (estrogen receptor positive) | Open-label, single-arm, one-way crossover pharmacokinetic study | Netherlands  (2023) | 26 (15 for primary pharmacokinetic endpoint) | Determine pharmacokinetic profile of tamoxifen when using CBD-oil and investigate if CBD-oil has beneficial effect on tamoxifen-related side effects | Tamoxifen (20-40 mg daily) + CBD-oil (≈50 mg daily for 4 weeks) | CBD-oil (good quality, <50 mg/day) does not significantly impact tamoxifen pharmacokinetics and may improve tamoxifen-related side effects. | [47] |
| Colorectal, uterine, ovarian | Non-randomized, controlled pilot trial | Denmark  (2023) | 32 (22 intervention, 10 control) | Investigate effect of cannabidiol (CBD) on lean body mass and taste alterations during chemotherapy | CBD 300 mg/day for 8 days per chemotherapy cycle | CBD may improve patients ability to differentiate taste strengths during chemotherapy (at baseline, the intervention group was unable to differentiate between the concentrations but gained the ability to significantly differentiate between weak and strong sweetness (p = 0.03) and weak and strong saltiness (p = 0.04) after three cycles of chemotherapy and treatment with CBD) | [48] |

Table S4 (Human Clinical Trials) evaluated using the Cochrane RoB 2 framework, and Table S5 (Preclinical *In Vivo* Animal Trials) evaluated using the SYRCLE tool.

Table S4 grades the 5 human clinical trials included in the manuscript across the five standard Cochrane domains, evaluating specific symptom or pharmacokinetic endpoints.

**Table S4. Cochrane RoB 2 Quality Matrix for Human Clinical Trials**

| **Study** | **D1**  **Randomization Process** | **D2**  **Deviations from Interventions** | **D3**  **Missing Outcome Data** | **D4**  **Outcome Measurement** | **D5**  **Selection of Results** | **Overall Risk of Bias Summary** |
| --- | --- | --- | --- | --- | --- | --- |
| Good et al. (2020) | Some Concerns  (Open-label pilot design) | Some Concerns  (Participants/caregivers unblinded) | Low Risk  (Small scale, cohort accounted for) | Some Concerns  (Subjective symptom distress scores) | Low Risk  (Predefined feasibility endpoints met) | Some Concerns  A valuable pilot study, but inherently limited by open-label parameters. |
| Hardy et al. (2023) | Low Risk  (Phase IIb robust randomization) | Low Risk  (Double-blind framework maintained) | Some Concerns  (Standard clinical dropout variance) | Low Risk  (Placebo-controlled blind scoring) | Low Risk  (Complete endpoint reporting for day 14) | Low Risk  High methodological quality with low risk of baseline distortion. |
| Gurgenci et al. (2023) | Low Risk  (Randomized controlled trial protocol) | Low Risk  (Blinded allocation tracking) | Some Concerns  (Palliative attrition not fully split) | Low Risk  (Objective anti-inflammatory lab targets) | Low Risk  (Explicit focus on non-detectable effect) | Low Risk  Strong experimental design with clear protective parameters against bias. |
| Buijs et al. (2023) | High Risk  (Single-arm, non-randomized) | Some Concerns  (Open-label execution) | Low Risk  (Pharmacokinetic data fully mapped) | Low Risk  (Objective mass spectrometry for tamoxifen) | Low Risk  (Primary pharmacokinetic goals met) | Some Concerns  High risk for subjective endpoints, but low risk for objective lab concentrations. |
| Dominiak et al. (2023) | High Risk  (Non-randomized assignment) | Some Concerns  (Unblinded allocation of intervention) | Low Risk  (Cohort completely tracked via cycles) | Some Concerns  (Subjective taste alteration values) | Low Risk  (Reported sweet/salty $p$-values completely) | Some Concerns  Methodological caution required due to non-randomized allocation layout. |

Table S5 scores the 7 preclinical animal models included in the manuscript across the 10 standard SYRCLE operational requirements. Scores are marked as Low (Low risk of bias), High (High risk of bias), or Unclear (reporting gap in original article).

**Table S5. SYRCLE Risk-of-Bias Matrix for Preclinical *In Vivo* Animal Trials**

| **Study** | **Q1**  **Sequence Generation** | **Q2**  **Baseline Characteristics** | **Q3**  **Allocation Concealment** | **Q4**  **Random Housing** | **Q5**  **Blind Care** | **Q6**  **Random Selection** | **Q7**  **Blind Assessment** | **Q8**  **Incomplete Data** | **Q9**  **Selective Report** | **Q10**  **Other Bias** |
| --- | --- | --- | --- | --- | --- | --- | --- | --- | --- | --- |
| **Romano et al. (2014)** | Unclear | Low  (ICR/Nude explicit) | Unclear | Unclear | Unclear | Unclear | Unclear | Unclear | Low  (Clear targets) | Unclear |
| **Singer et al. (2015)** | Unclear | Low  (Athymic nu/nu mice) | Unclear | Unclear | Unclear | Unclear | Unclear | Unclear | Low  (ROS/GSC tracked) | Unclear |
| **Fisher et al. (2016)** | Unclear | Low  (NOD/SCID models) | Unclear | Unclear | Unclear | Unclear | Unclear | Unclear | Low  (Apoptosis metrics) | Unclear |
| **Ferro et al. (2018)** | Unclear | Low  (KPCG strain target) | Unclear | Unclear | Unclear | Unclear | Unclear | Unclear | Low  (GPR55 outcome) | Unclear |
| **Pongking et al. (2024)** | Unclear | Low  (BALB/c Nu/Nu lines) | Unclear | Unclear | Unclear | Unclear | Unclear | Unclear | Low  (ER-stress focus) | Unclear |
| **Wang et al. (2024)** | Unclear | Low  (C57BL/6 standard) | Unclear | Unclear | Unclear | Unclear | Unclear | Unclear | Low  (TIAR-1 metrics) | Unclear |
| **Mangal et al. (2024)** | Unclear | Low  (FVB/NJ female line) | Unclear | Unclear | Unclear | Unclear | Unclear | Unclear | Low  (CerS1 activation) | Unclear |

References

1.	A. Ligresti et al., *Antitumor activity of plant cannabinoids with emphasis on the effect of cannabidiol on human breast carcinoma.* The Journal of pharmacology experimental therapeutics, 2006. 318(3): p. 1375-1387.

2.	L. De Petrocellis et al., *Non‐THC cannabinoids inhibit prostate carcinoma growth in vitro and in vivo: pro‐apoptotic effects and underlying mechanisms.* British journal of pharmacology, 2013. 168(1): p. 79-102.

3.	K. A. Scott et al., *Enhancing the activity of cannabidiol and other cannabinoids in vitro through modifications to drug combinations and treatment schedules.* Anticancer Research, 2013. 33(10): p. 4373-4380.

4.	B. Romano et al., *Inhibition of colon carcinogenesis by a standardized Cannabis sativa extract with high content of cannabidiol.* Phytomedicine, 2014. 21(5): p. 631-639.

5.	T. Macpherson et al., *Physiological intestinal oxygen modulates the Caco-2 cell model and increases sensitivity to the phytocannabinoid cannabidiol.* In Vitro Cellular Developmental Biology-Animal, 2014. 50: p. 417-426.

6.	K. A. Scott et al., *The combination of cannabidiol and Δ9-tetrahydrocannabinol enhances the anticancer effects of radiation in an orthotopic murine glioma model.* Molecular cancer therapeutics, 2014. 13(12): p. 2955-2967.

7.	E. Singer et al., *Reactive oxygen species-mediated therapeutic response and resistance in glioblastoma.* Cell death disease, 2015. 6(1): p. e1601-e1601.

8.	T. S. Rajan et al., *Anti-inflammatory and antioxidant effects of a combination of cannabidiol and moringin in LPS-stimulated macrophages.* Fitoterapia, 2016. 112: p. 104-115.

9.	T. Fisher et al., *In vitro and in vivo efficacy of non-psychoactive cannabidiol in neuroblastoma.* Current oncology, 2016. 23(Suppl 2): p. S15.

10.	S. T. Lukhele et al., *Cannabidiol rather than Cannabis sativa extracts inhibit cell growth and induce apoptosis in cervical cancer cells.* BMC complementary alternative medicine, 2016. 16: p. 1-16.

11.	N. Kalenderoglou et al., *Cannabidiol reduces leukemic cell size–but is it important?* Frontiers in Pharmacology, 2017. 8: p. 144.

12.	R. Ferro et al., *GPR55 signalling promotes proliferation of pancreatic cancer cells and tumour growth in mice, and its inhibition increases effects of gemcitabine.* Oncogene, 2018. 37(49): p. 6368-6382.

13.	W. M. Raup-Konsavage et al., *Synthetic cannabinoid activity against colorectal cancer cells.* Cannabis cannabinoid research, 2018. 3(1): p. 272-281.

14.	B. M. Fonseca et al., *Cannabinoid-induced cell death in endometrial cancer cells: involvement of TRPV1 receptors in apoptosis.* Journal of physiology biochemistry, 2018. 74(2): p. 261-272.

15.	M. Olivas-Aguirre et al., *Cannabidiol directly targets mitochondria and disturbs calcium homeostasis in acute lymphoblastic leukemia.* Cell death disease, 2019. 10(10): p. 779.

16.	A. Sainz-Cort et al., *Anti-proliferative and cytotoxic effect of cannabidiol on human cancer cell lines in presence of serum.* BMC research notes, 2020. 13(1): p. 389.

17.	M. Mazuz et al., *Synergistic cytotoxic activity of cannabinoids from cannabis sativa against cutaneous T-cell lymphoma (CTCL) in-vitro and ex-vivo.* Oncotarget, 2020. 11(13): p. 1141.

18.	A. I. Fraguas-Sánchez et al., *CBD loaded microparticles as a potential formulation to improve paclitaxel and doxorubicin-based chemotherapy in breast cancer.* International journal of pharmaceutics, 2020. 574: p. 118916.

19.	W. M. Raup-Konsavage et al., *Cannabidiol (CBD) oil does not display an entourage effect in reducing cancer cell viability in vitro.* Medical Cannabis Cannabinoids, 2020. 3(2): p. 95-102.

20.	N. Patel et al., *Cannabidiol loaded extracellular vesicles sensitize triple-negative breast cancer to doxorubicin in both in-vitro and in vivo models.* International journal of pharmaceutics, 2021. 607: p. 120943.

21.	C. Gross et al., *Cannabidiol induces apoptosis and perturbs mitochondrial function in human and canine glioma cells.* Frontiers in Pharmacology, 2021. 12: p. 725136.

22.	R. F. Maguire et al., *The pharmacological effects of plant-derived versus synthetic cannabidiol in human cell lines.* Medical Cannabis Cannabinoids, 2021. 4(2): p. 86-96.

23.	S. K. Surapaneni et al., *Anticancer and chemosensitization effects of cannabidiol in 2D and 3D cultures of TNBC: Involvement of GADD45α, integrin-α5,-β5,-β1, and autophagy.* Drug delivery translational research, 2022. 12(11): p. 2762-2777.

24.	C. Andradas et al., *Assessment of cannabidiol and Δ9-tetrahydrocannabiol in mouse models of medulloblastoma and ependymoma.* Cancers, 2021. 13(2): p. 330.

25.	P. Zhu et al., *Self-assembly system based on cyclodextrin for targeted delivery of cannabidiol.* Frontiers in Chemistry, 2021. 9: p. 754832.

26.	A. M. Tomko et al., *Anti-cancer properties of cannabidiol and Δ9-tetrahydrocannabinol and potential synergistic effects with gemcitabine, cisplatin and other cannabinoids in bladder cancer.* bioRxiv, 2021: p. 2021.03. 25.436633.

27.	L. Anceschi et al., *Chemical characterization of non‐psychoactive Cannabis sativa L. extracts, in vitro antiproliferative activity and induction of apoptosis in chronic myelogenous leukaemia cancer cells.* Phytotherapy Research, 2022. 36(2): p. 914-927.

28.	T. Tajik et al., *Extracellular vesicles of cannabis with high CBD content induce anticancer signaling in human hepatocellular carcinoma.* Biomedicine Pharmacotherapy, 2022. 152: p. 113209.

29.	T. Lamtha et al., *Structural analysis of cannabinoids against EGFR-TK leads a novel target against EGFR-driven cell lines.* Current Research in Pharmacology Drug Discovery, 2022. 3: p. 100132.

30.	A. D’Aloia et al., *Cannabidiol antiproliferative effect in triple-negative breast cancer MDA-MB-231 cells is modulated by its physical state and by IGF-1.* International journal of molecular sciences, 2022. 23(13): p. 7145.

31.	R. Razlog et al., *Cytotoxic effects of combinative ZnPcS4 photosensitizer photodynamic therapy (PDT) and cannabidiol (CBD) on a cervical cancer cell line.* International journal of molecular sciences, 2023. 24(7): p. 6151.

32.	P. Śledziński et al., *In vitro evidence of selective pro-apoptotic action of the pure cannabidiol and cannabidiol-rich extract.* Molecules, 2023. 28(23): p. 7887.

33.	E. G. Whynot et al., *Anticancer properties of cannabidiol and Δ9-tetrahydrocannabinol and synergistic effects with gemcitabine and cisplatin in bladder cancer cell lines.* Journal of Cannabis Research, 2023. 5(1): p. 7.

34.	X. Sun et al., *Single-cell analyses reveal cannabidiol rewires tumor microenvironment via inhibiting alternative activation of macrophage and synergizes with anti-PD-1 in colon cancer.* Journal of pharmaceutical analysis, 2023. 13(7): p. 726-744.

35.	A. Štern et al., *Exploring the safety of cannabidiol (CBD): A comprehensive in vitro evaluation of the genotoxic and mutagenic potential of a CBD isolate and extract from Cannabis sativa L.* Biomedicine Pharmacotherapy, 2024. 177: p. 116969.

36.	S. Ceccacci et al., *A label free chemoproteomic-based platform to disclose cannabidiol molecular mechanism of action on chronic myelogenous leukemia cancer cells.* Heliyon, 2024. 10(1).

37.	T. Pongking et al., *Cannabidiol exhibits potent anti-cancer activity against gemcitabine-resistant cholangiocarcinoma via ER-stress induction in vitro and in vivo.* BMC Complementary Medicine Therapies, 2024. 24(1): p. 325.

38.	N. Mangal et al., *Cannabidiol’s cytotoxicity in pancreatic cancer is induced via an upregulation of ceramide synthase 1 and ER stress.* Journal of Cannabis Research, 2024. 6(1): p. 22.

39.	L. P. Wang et al., *Altering biomolecular condensates as a potential mechanism that mediates cannabidiol effect on glioblastoma.* Medical Oncology, 2024. 41(6): p. 140.

40.	F. Mottarlini et al., *Single and repeated exposure to cannabidiol differently modulate BDNF expression and signaling in the Cortico-Striatal Brain Network.* Biomedicines, 2022. 10(8): p. 1853.

41.	Z. Wang et al., *Cannabidiol induces autophagy and improves neuronal health associated with SIRT1 mediated longevity.* Geroscience, 2022. 44(3): p. 1505-1524.

42.	R. G. Henderson et al., *Genotoxicity evaluation of cannabidiol.* Regulatory Toxicology Pharmacology, 2023. 142: p. 105425.

43.	M. J. Tallon et al., *Subchronic oral toxicity assessment of a cannabis extract.* Regulatory Toxicology Pharmacology, 2023. 144: p. 105496.

44.	P. D. Good et al., *An open-label pilot study testing the feasibility of assessing total symptom burden in trials of cannabinoid medications in palliative care.* Journal of palliative medicine, 2020. 23(5): p. 650-655.

45.	J. Hardy et al., *Phase IIb randomized, placebo-controlled, dose-escalating, double-blind study of cannabidiol oil for the relief of symptoms in advanced cancer (MedCan1-CBD).* Journal of Clinical Oncology, 2023. 41(7): p. 1444-1452.

46.	T. Gurgenci et al., *Exploring potential anti-inflammatory effects of medicinal cannabis.* Supportive Care in Cancer, 2023. 31(11): p. 629.

47.	S. M. Buijs et al., *CBD-oil as a potential solution in case of severe tamoxifen-related side effects.* npj Breast Cancer, 2023. 9(1): p. 63.

48.	H. S. Dominiak et al., *Prevention of taste alterations in patients with cancer receiving Paclitaxel-or Oxaliplatin-based chemotherapy—A pilot trial of cannabidiol.* Nutrients, 2023. 15(13): p. 3014.
